# Multiscale, mechanistic model of Rheumatoid Arthritis to enable decision making in late stage drug development

**DOI:** 10.1038/s41540-024-00454-1

**Published:** 2024-11-04

**Authors:** Dinesh Bedathuru, Maithreye Rengaswamy, Madhav Channavazzala, Tamara Ray, Prakash Packrisamy, Rukmini Kumar

**Affiliations:** Vantage Research Inc, Lewes, Lewes, DE USA

**Keywords:** Immunology, Numerical simulations

## Abstract

Rheumatoid Arthritis (RA) is a chronic autoimmune inflammatory disease that affects about 0.1% to 2% of the population worldwide. Despite the development of several novel therapies, there is only limited benefit for many patients. Thus, there is room for new approaches to improve response to therapy, including designing better trials e.g., by identifying subpopulations that can benefit from specific classes of therapy and enabling reverse translation by analyzing completed clinical trials. We have developed an open-source, mechanistic multi-scale model of RA, which captures the interactions of key immune cells and mediators in an inflamed joint. The model consists of a treatment-naive Virtual Population (Vpop) that responds appropriately (i.e. as reported in clinical trials) to standard-of-care treatment options—Methotrexate (MTX) and Adalimumab (ADA, anti-TNF-α) and an MTX inadequate responder sub-population that responds appropriately to Tocilizumab (TCZ, anti-IL-6R) therapy. The clinical read-outs of interest are the American College of Rheumatology score (ACR score) and Disease Activity Score (DAS28-CRP), which is modeled to be dependent on the physiological variables in the model. Further, we have validated the Vpop by predicting the therapy response of TCZ on ADA Non-responders. This paper aims to share our approach, equations, and code to enable community evaluation and greater adoption of mechanistic models in drug development for autoimmune diseases.

## Introduction

RA is an autoimmune disease i.e., a disease which arises due to an abnormal immune reaction of the body against a part of itself; in this case the soft tissue and bones of joints, affecting 0.1 -0.2% of people worldwide^1,2^. Treatments include broad-spectrum anti-inflammatory agents (Steroids, Non-Steroidal Anti-Inflammatory Drugs), disease-modifying antirheumatic drugs (DMARDS, such as Methotrexate), followed by targeted antibodies (such as anti-TNF-α) and novel small molecules which target intracellular signaling pathways (such as JAK inhibitors). RA can cause severe inflammation of the joints, leading to bone erosion, overgrowth of synovial tissue (or pannus) and loss of mobility in the joints over time, in addition to extreme pain.

According to the guidelines of the American College of Rheumatology (ACR), Disease-Modifying Antirheumatic Drugs (DMARDs) are recommended as the first line of therapy against RA to minimize the damage to the joints and increase chances of remission of the disease^3–5^. These include conventional DMARDs such as methotrexate, hydroxychloroquine or sulfasalazine and biological DMARDS such as anti-TNF-α, anti-IL-6R, anti-CD20, kinase inhibitors like Tofacitinib etc.^6^ The goal of RA therapy is to achieve remission of the disease and prevent progression of bone damage. However, there is large variability between patients with respect to their response to therapy. Often, the majority of patients do not achieve remission and even those who respond may lose the response to therapy over time and need higher doses or new therapies or combinations^7,8^. Hence, there is a need to understand the underlying mechanisms of response to therapy and development of resistance so that effective dosing regimens and combinations can be determined and potentially personalized to the patient^9^.

Quantitative Systems Pharmacology (QSP) enables the efficient development of novel therapies by integrating data and knowledge quantitatively across multiple scales. We have developed a QSP model of the key interactions of immune mediators within an inflamed joint that can predict therapeutic combinations, impact of protocol variations (identifying the right dose/combination/regimen), and potentially identification of patient types so that the intervention benefits the most patients. The scope of this study is to enable simulation of stable RA disease in a drug development context for Phase 2/Phase 3 clinical trials to evaluate between therapeutic agents. Other aspects of interest that have been excluded in the current scope are capturing the onset and progression of disease, flares and personalized patient-related details such as age, gender and prior treatment. However, the model is shared publicly, and its structure allows for such modifications by other interested groups.

## Results

We have developed a model of stable RA disease, which includes key immune cells and mediators in an inflamed joint and captures clinical endpoints reported for a population of RA patients^10,11^. We have followed the general approach to develop QSP models as described in the literature and have illustrated our development process in steps in the Methods section, that correspond to those in Gadkar et al. ^12–14^.

### Model design and parameterization

**Fig. 1:**
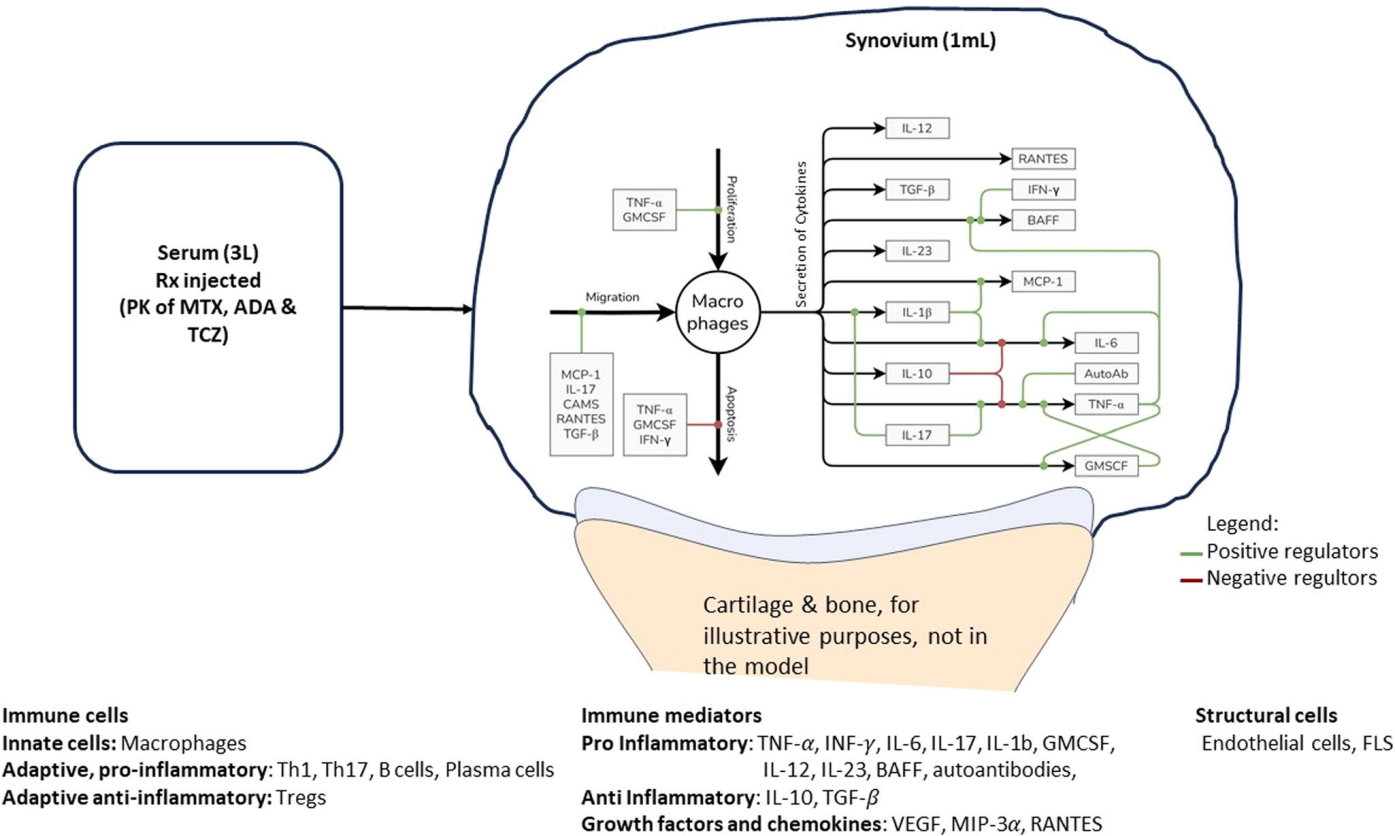
Model compartments and components. The synovial compartment is the site of the inflammation with several immune cells, mediators, and structural cells present. The cells secrete multiple pro and anti-inflammatory cytokines, chemokines, and growth factors as shown in the figure (Macrophages as an exemplar). All the cells in the synovium undergo proliferation and apoptosis in the synovial compartment. Immune cells can also migrate into the synovium from the serum while FLS does not. The serum compartment is the source of immune cells and therapeutics in synovium. The life cycle and regulation of one representative cell type in the model is shown here with regulators of proliferation, migration, apoptosis, and cytokine secretion. The life cycle of the other cells in the model and their regulators shown in Fig. 7.

**Fig. 2:**
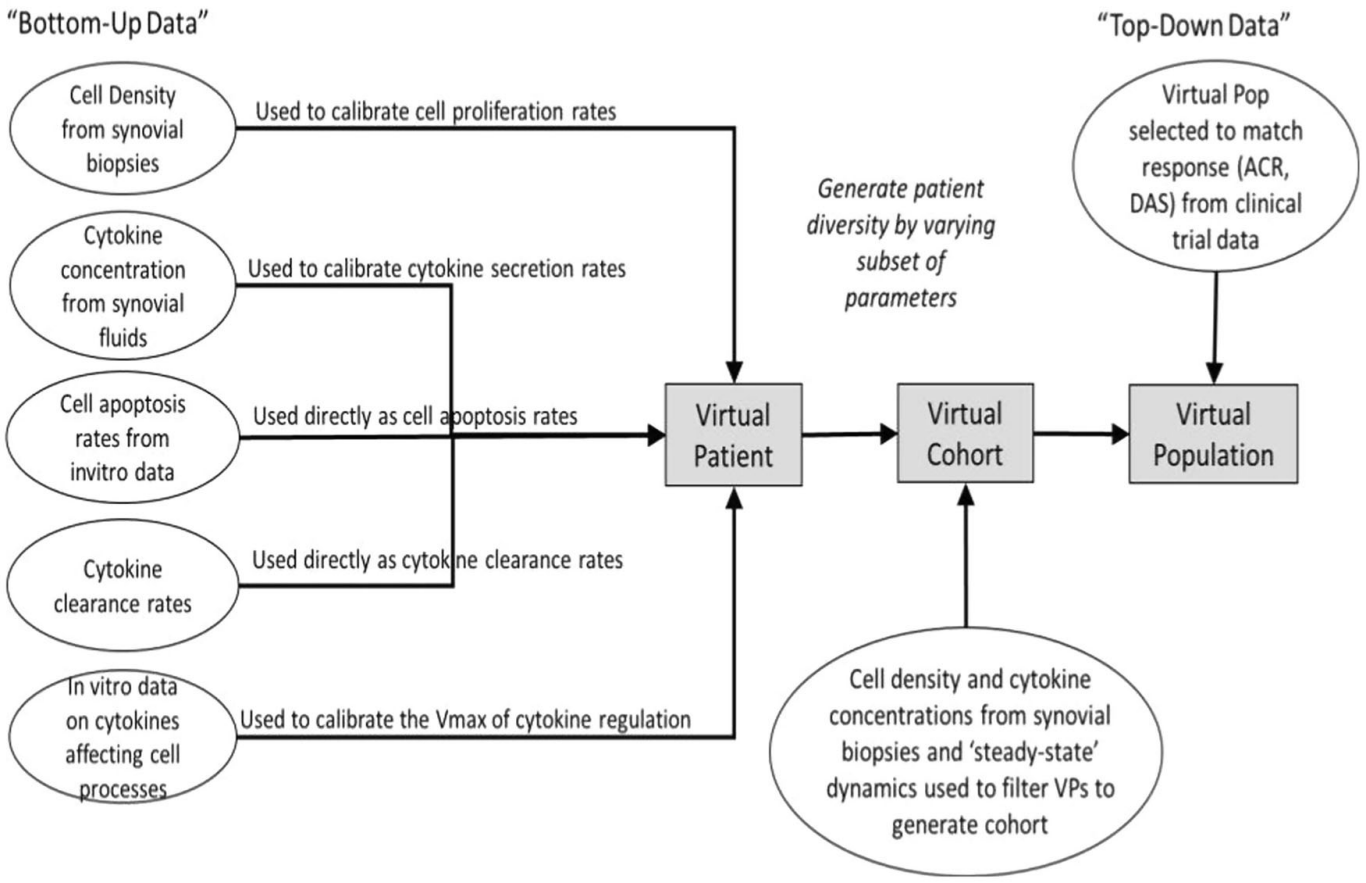
The workflow to go from model equations to a RA Vpop. Once the equations are set up, based on a survey of literature, some cell life cycle and cytokine clearance rates have been fixed. Other parameters are estimated to match dynamic steady-state values of cells reported. Once a Reference Virtual Patient at steady state with physiologically reasonable parameters has been developed, a subset of parameters is varied to create a Virtual Cohort of plausible VPs. Based on their response to therapy, a Vpop that responds appropriately to therapies of interest is selected from the Virtual Cohort.

The site of inflammation in RA is the joint, consisting of bone, cartilage, synovial membrane, and synovial fluid. Of these, the synovial membrane is the key site of inflammation with several immune cells and cytokines present^15^. We have assumed a well-mixed volume of 1 mL of synovium to be the site of interest to capture the basic pathophysiology of the disease. Inflammatory cells, cytokines and structural cells interact in our idealized synovial volume (Fig. 1 shows the modeling of Macrophages. Similar figures for all cell types and cytokines are available in Fig. 7). Once the model design process (more details in the Methods section) has resulted in the effect diagram and a set of equations for the model, the next step is determining the quantitative ranges of the parameters of the model. The broad approach is shown in Fig. 2, where “bottom-up” data from in vitro and preclinical experiments have been used to inform parameters and “top-down” data from clinical trials have been used to constrain the Vpop. We will focus on the Vpop in the Results and Discussion, but more details on model quantification have been discussed in the Methods section.

### A single Calibrated Virtual Population consistent with available MTX, ADA and TCZ monotherapies was obtained

**Fig. 3:**
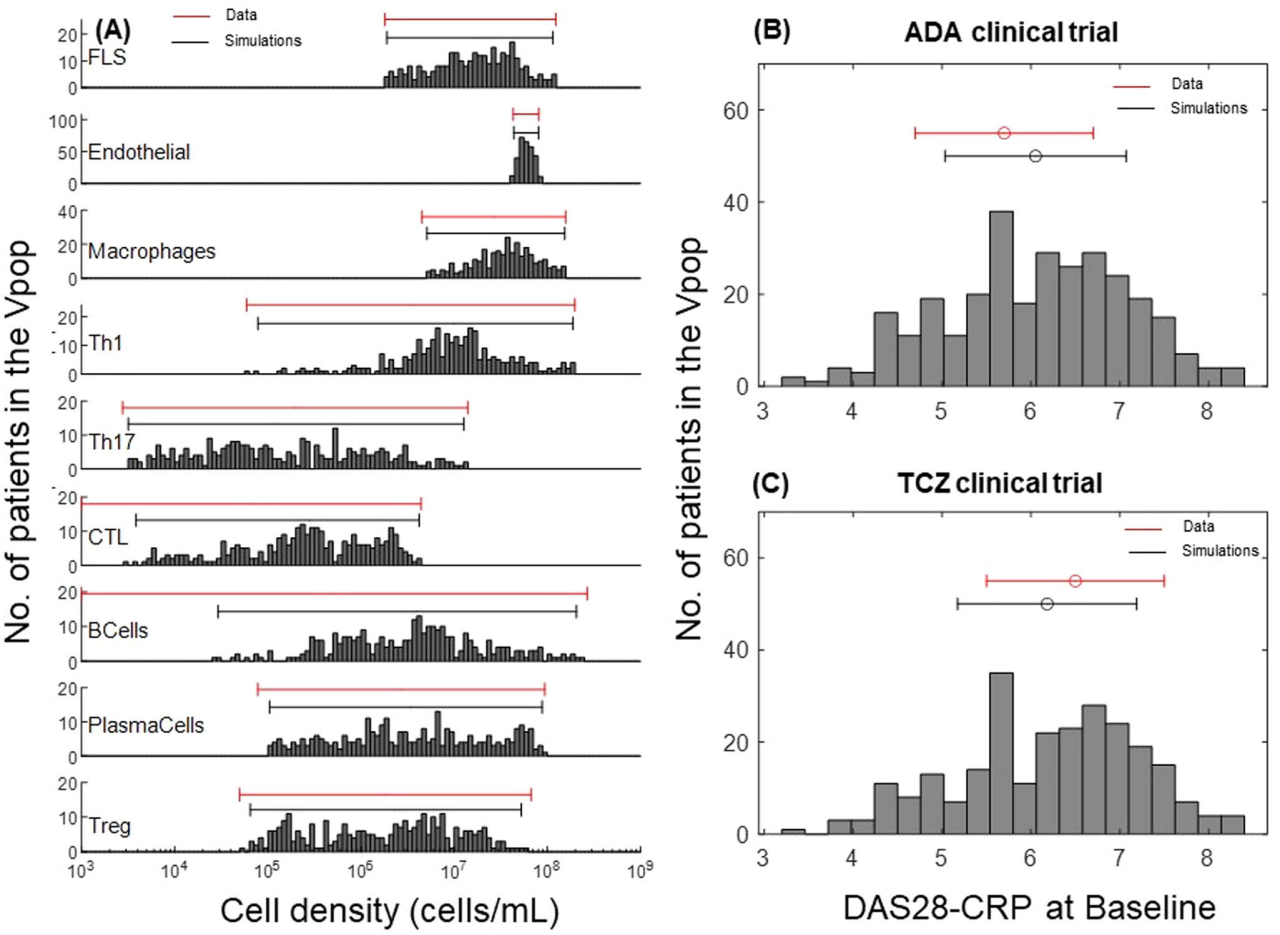
Distribution of cell densities and clinical scores at baseline in the Vpop. **A** Comparison of **baseline distributions** of the synovial cell densities of the Vpop with the ranges from public literature. The histogram shows the distribution of cell densities in the Vpop while the red line above indicates the range of cell densities of different cell types obtained from literature. **B** and **C** Comparison of DAS28-CRP histogram of the Vpop at baseline with the reported mean and standard deviation from the clinical trials for ADA (**B**) and TCZ (**C**) clinical trials. The red and black bars denote the values reported in the trials and the simulated Vpop outcome, respectively.

As described in the Methods section, we developed the model, quantified it, and generated a Vpop which is at dynamic ‘steady state’ with respect to the cells and cytokines represented in the model for various levels of severity of RA disease. Figure 3 shows the synovial cell densities of different cell types at baseline and in comparison, to reported ranges in the literature. (See Supplementary Data 1 for the literature from which these ranges were derived) The Vpop (*n* = 300) is well distributed within these ranges. We selected three therapies - Methotrexate (MTX), Adalimumab (ADA) and Tocilizumab (TCZ) to calibrate the Vpop from the Virtual Cohort, as discussed in the Methods. All patients in the Vpop were entered into the MTX and ADA trials and the subset of Virtual patients in this trial, which were non-responders to MTX (*n* = 251) were put on TCZ. Figure 3 shows the distribution of DAS28-CRP scores of the VPop at baseline, along with reported mean and standard deviation at baseline from the Kavanaugh et al. ^16^ and Yazici et al. ^17^ trials on ADA and TCZ respectively^16,17^ (See Supplementary Data 1 for the detailed representation of available data from these trials). (The Strand et al. ^18^ study on MTX did not report baseline DAS28-CRP)^18^. The approach to derive disease scores from synovial densities is elaborated in the Methods section, under ‘Model parameterization: Implementation of disease severity scores’.

**Fig. 4:**
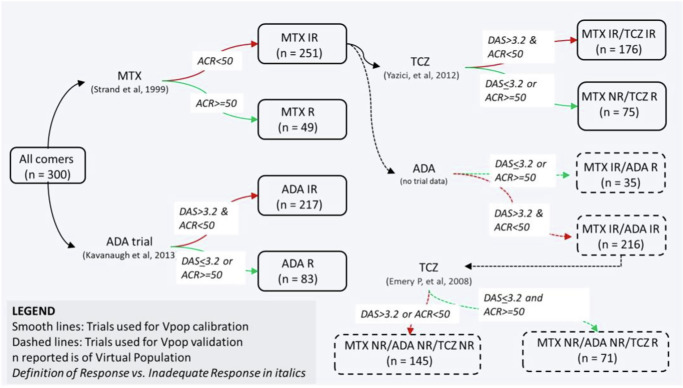
The Vpop and the order in which MTX, ADA and TCZ were simulated. Initially all comers were administered MTX and ADA monotherapy and the VPop was calibrated to be consistent with both. Then, MTX IRs were administered TCZ and the subset of MTX IRs were also consistent with TCZ monotherapy. The VPop was validated against Emery P. et al. ^20^ which administered TCZ to MTX & ADA IRs and without any further adjustment, was able to fit the data. The smooth lines of the figure show the trials used to calibrate the VPop and the dotted lines were validation where no further edits were made and the fit of the Vpop to the trial was examined. The definition of IR was not always explicit in the papers. When this definition was not mentioned, we defined IR as stated in the figure.

We implemented the PK and PD of the various interventions of interest - MTX, ADA and TCZ monotherapies as explained in the Methods section. In particular, the patients entering the MTX and ADA trials were “all comers” (i.e., not previously treated by either therapy but may have been treated by NSAIDS), whereas the TCZ trial was on MTX Inadequate Responders (IR). We simulated the trials as described starting with a single Vpop that is consistent with all the reported clinical scores reported (Fig. 4).

**Fig. 5:**
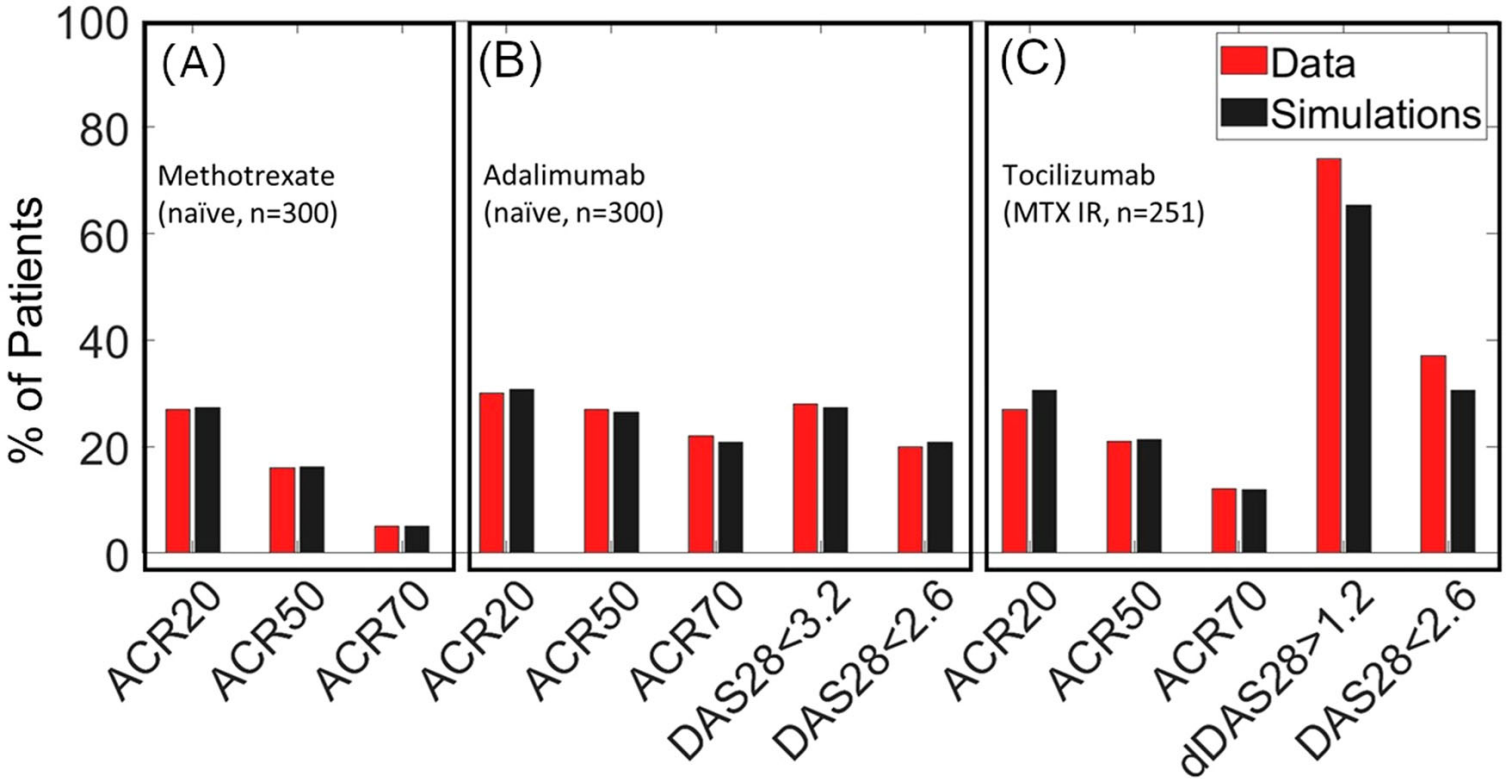
The calibrated response of the Vpop to MTX, ADA and TCZ. The calibrated response of the Vpop to MTX and ADA (**A**) MTX (Week 12), (**B**) ADA (Week 24 for ACR and Week 26 for DAS28-CRP) and (**C**) TCZ (Week 24), showing a good match to the trial data in terms of reported DAS and ACR scores. Simulation conditions are taken from published clinical trials protocols as reported in the Methods. ‘Data’ refers to the clinical trial outcomes after placebo correction. TCZ clinical trial was run on MTX inadequate responder population, hence the Vpop is calibrated such that its MTX IR subpopulation (ACR < 50% and DAS28-CRP > 3.2 post therapy) matches the clinical trial outcome.

Figure 5 shows the results of calibration to therapy in terms of the metrics reported for clinical efficacy—ACR20, 50 and 70, as well as DAS28-CRP < 3.2 DAS28-CRP < 2.6. DAS28-CRP and dDAS28-CRP > 1.6. As can be seen from the figure, the Vpop calibration is a close match to data both for the MTX naive population and for the MTX-IR population used for calibration of TCZ. We also show that the distribution of DAS28-CRP scores in the VPop for each of these trials (see Fig. 11) shows sufficient phenotypic diversity in the response. The representation of DAS28-CRP scores in the model is discussed in the section “Additional information on methods, 4. Representing DAS28-CRP scores”. There can be tradeoffs in how to determine the Vpop fit, our goal was to have a range of poor and strong responders and thus directed our Vpop development accordingly.

An additional consideration in interpreting clinical scores in auto-immune trials is factoring in the response of the placebo arm in the trial. The efficacy attributable to the drug should be the placebo efficacy ‘subtracted’ from the reported efficacy. There are several approaches to implement placebo correction and we have followed the one recommended by Wang et al. ^19^, which is described in greater detail in the Methods section, under Stage 4 and 5^19^.

### Validation: Predicting the outcome of a Tocilizumab trial on anti-TNF-α non-responder population

**Fig. 6:**
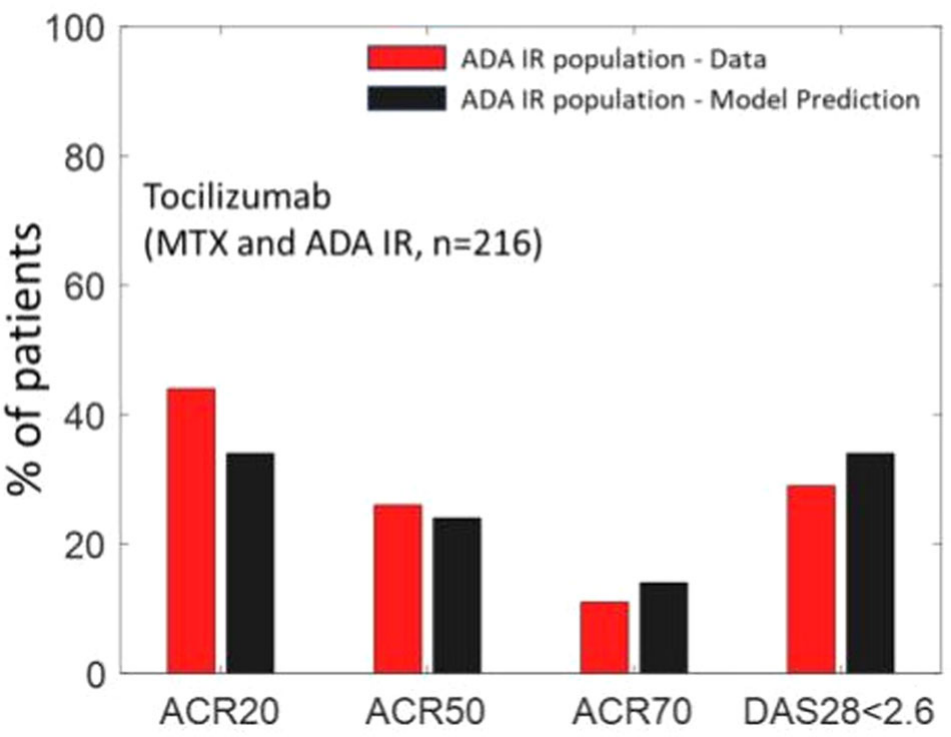
Prediction of ACR categories and DAS28-CRP scores on TCZ therapy at week 24 for a dosing regimen of 8 mg/kg at Q4W, on a subpopulation which is ADA and MTX IR. The results were validated against a clinical trial of patients with similar characteristics i.e. with ADA and MTX IR patients^20^. The fractions of patients which are ACR 50, ACR 70 and DAS28-CRP < 2.6 is within 5% of reported values.

Model validation increases the confidence in the model – typically this is carried out by predicting impact of interventions which the model has not been calibrated to. This can be carried out by predicting the efficacy of novel therapies, combinations, or impact of calibrated therapies in new subpopulations. We validated our model by predicting the impact of Tocilizumab on a sub-population of MTX and ADA inadequate responders (IRs). This subpopulation (of *n* = 216) was selected as the set of Virtual patients with ACR < 50 and DAS28-CRP > 3.2 post-therapy for MTX and ADA. Figure 6 shows the outcome of our model simulations compared to the data obtained from a clinical trial on the efficacy of TCZ in such an ADA + MTX IR population of patients^20^. As seen from Fig. 6, the predicted reduction in DAS28-CRP and ACR scores, with no further adjustments, using the calibrated Vpop shows a good match to ACR50, ACR70 and DAS28-CRP remission (i.e., DAS28-CRP < 2.6) scores. The match is poorer for ACR 20 though within 10%.

## Discussion

In this paper, we have developed a QSP model of Rheumatoid Arthritis that connects key immune players in an inflamed joint to clinical scores observed in multiple trials. Academic models of RA have often focused on specific aspects of RA disease – for example, progression of the disease was analyzed by a general model of erosion of tissue and a more complex spatial model captures the movement of the boundary between the synovium and cartilage due to immune interactions^21–23^. QSP models of RA have also been used in pharmaceutical companies to aid decision making e.g., the Entelos RA Physiolab platform and the Rosa PhysioPD platforms were used to predict the efficacy of novel therapies in clinical trials, in the selection of suitable biomarkers, and to understand the mechanistic basis of variability in response to therapy etc. ^24–27^ However, these models are not publicly available and therefore further evaluation and research using them is not possible.

Here, we have published a calibrated and validated model of RA and discuss how this model can be further used to provide a mechanistic understanding of the pathogenesis of RA and patient heterogeneity. The Vpop that we have developed can also be interrogated further such as to test combinations therapy (for example, we showed MTX + ADA from an earlier version of the model), test alternate dosing regimens and visualize trial designs^10^. The model can be improved for RA by the addition of other immune players of interest or other biomarkers (such as CRP). Further, auto-immune diseases are similar and such a model can be adapted to other diseases such as IBD, Psoriasis etc. ^13^

However, there are limitations with our approach that can be improved upon. We have calibrated based on mean and variance of publicly available data, this can be further refined when proprietary individual patient data is available to sponsors. In particular, recent studies have used “digital twins” to match multiple Virtual Patients to a single real patient to track their particular trajectory^28^. Another area that can be improved with availability of individual patient data is the connection to the disease score. At this time, we have used a theoretical link between the density of cells in the synovium to predict disease scores and while this is reasonable given the available public data, this can be improved when tissue biopsy, multi-omics data etc. is available to connect every patient to their disease score.

QSP models that integrate knowledge and data from multiple scales and sources, quantitatively, that enable better decision-making in drug development can be very powerful tools. This is especially true in auto-immune diseases where there are multiple cells and mediators, multiple therapies and diversity in patient response. In this case, having such a modeling framework can be used to test hypotheses and predict outcomes and improve odds of success. QSP models that enable asking what-if questions (prospective simulations) allow for better experimental design and interpretation of clinical data to refine our understanding.

Published models can enable greater adoption of approaches like QSP as the model can be examined by many in the scientific community^29,30^. In all analyses and especially in QSP, modeler choices are critical aspects that can affect how to interpret the model recommendations. There are often trade-offs in all choices, but by being transparent about them, we are enabling the recommendation to be interrogated and further improved.

## Methods

### Stage 1: Establishing Project Goals

Our objective was to develop a model that could be used for the simulation of late-stage clinical trials (induction of Phase 2/3) that captures quantitatively the dynamics of cells and mediators in a mechanistic physiological scale and the impact of the therapy on the physiology and its translation to clinical disease activity scores within a time scale of a few months. We created a Vpop with varying disease severity of established, stable disease (vs. preclinical or early disease based on varying levels of cells and mediators in the joint)^31^. This Vpop is calibrated to be consistent with disease activity scores at baseline and in response to methotrexate and ADA, as reported in clinical trials^16–18,32^. We aimed to develop a model structure that can add new biology and targets of interest within this framework; future versions will build on this scope and add functionality and be compatible with the features presented here.

### Stage 2: Designing Model Scope and Complexity

The model design is as simple as necessary to capture the disease pathophysiology relevant to simulating the induction and maintenance phases of a typical Phase 2 or Phase 3 clinical trial, which runs for a few months in patients with established RA^29^. The model is limited to capturing established steady-state disease (which can be mild, moderate, or severe) and does not capture the poorly understood complexities of disease onset, flares, and disease progression over several years^15,33^.

The site of inflammation in RA is the joint, consisting of bone, cartilage, synovial membrane, and synovial fluid. Of these, the synovial membrane is the key site of inflammation with several immune cells and cytokines present^15^. We have assumed a well-mixed volume of 1 mL synovium to be the site of interest to capture the basic pathophysiology of the disease. Inflammatory cells, cytokines and structural cells interact in our idealized synovial volume.

The two types of cells considered in the synovial compartment of the model are structural and immune cells. In the synovial tissue, cell-cell contact-mediated effects, which may contribute to disease progression, are not considered at this time and all effects are assumed to occur via cytokines^34^. The synovial volume itself is fixed over time in the model and the severity of disease is approximated by the cell density in the volume (higher for more severe disease).

Currently, other physiological components of interest, such as bone and cartilage are not captured in the model. Outside of the joint, we have a generalized plasma compartment. The lymph node is not modeled explicitly, and differentiated cells are assumed to migrate to synovial tissue from plasma. The model also does not consider heterogeneity between joints in a patient i.e., all joints of the patient are identical in their pathophysiology and response to therapy and only a single average joint is modeled. Cells and mediators involved in disease onset (such as Dendritic cells) and cells involved in other aspects of disease progression (like Osteoclasts involved in Bone erosion) were not captured in this version of the model. Autoantigen is not explicitly modeled, and we assume that excess autoantigen is always available (an argument for this approach is that tolerogenic therapies targeting autoantigen for RA have not shown much success^35^.

A detailed description of the different cell types and cytokines in the synovial compartment of the model is provided below:

A. Structural cells

- **Fibrocytes like Synoviocytes (FLS)**: This is a specialized cell type located inside joints in the synovium and it is critical in the pathophysiology of RA disease. The inflamed synovium results in the formation of Pannus which is considered the characteristic feature of RA, and FLS are the most common cell type at the pannus–cartilage junction and contribute to joint destruction through their production of cytokines, chemokines, and matrix-degrading molecules and by invading joint cartilage^30,36^.
- **Endothelial cells (E)**: Endothelial cells play an important role in many diseases of chronic inflammation. In RA they express adhesion molecules and present chemokines, which leads to leukocyte migration from the blood into the tissue. Angiogenesis is another characteristic feature of RA and proliferating ECs lead to more blood flow and that leads to more trafficking of immune cells to the inflamed joint^37^.

B. Immune cells and Cytokines

In general, the inflamed synovium is characterized by infiltration of multiple immune cells and increased cytokine concentration in comparison with healthy tissue^38^. At this time, we have included cells and mediators whose role in the disease is well-established and which have been considered for targeted therapies.

- **Macrophages**: Among the cells of the innate immune system, Macrophages are modeled. They are represented as a single homogeneous population, secreting both pro and anti-inflammatory cytokines. There is evidence that indicates greater complexity in the macrophages with multiple phenotypes in the inflamed RA joint, but this distinction may be made in later versions^38^.
- **Multiple Pro-Inflammatory Cells of the Adaptive Immune System**: The cells of the adaptive immune system are central contributors to inflammation in RA^39^. We have represented CD8 T Cells, Th1 and Th17 Cells, B Cells, and Plasma Cells.
- **CD4 Treg**: In RA, the role of anti-inflammatory cells is less well understood than that of pro-inflammatory cell types^40^. However, Tregs are a key player that can induce strong mediator induced effects that may affect the dynamics of the other cell types^41^.
- **Multiple Proinflammatory cytokines**: Inflammation in RA is assumed to be primarily promoted by the pro-inflammatory cytokines which are numerous and often have redundant functionality^42,43^. Several have been targeted in developing therapies with varying success. It is possible that the relative importance of cytokines may depend on the particular patient and their stage in disease progression^31^. We have included the following typical pro-inflammatory cytokines whose role in synovial inflammation has been studied and whose suppression has been successful in alleviating symptoms of stable RA disease in multiple studies: TNF-α, IL-6, IL-17, IL-12, and IL-23.
  Further, we have modeled IFN- γ and IL-1β as prototypical cytokines that have been shown to be important in establishing chronic inflammation. Some of their activity may be redundant with other cytokines, such as TNF-α, but their activity may also be necessary to incorporate as a ‘ceiling’ to the effectiveness of other anti-cytokine therapies. However, anti-IL-1β and anti-IFN-γ monotherapy have not been successful in mitigating symptoms of RA^44^. GMCSF may affect disease via the activation of macrophages and has been modeled. Recent studies have shown some promise for anti-GMCSF therapy in mono or combination in alleviating symptoms of RA^45^.
  B Cell Activating Factor, BAFF has been modeled to enable modulation effect on disease of B cell lineage^46^. Auto-antibodies are also modeled and are considered to be biomarkers of inflammation in some patients as well to play an important role in cytokine secretion by immune cells^47^. However, their impact on non-cytokine pathways (such as bone erosion, cartilage degradation) are not present. Thus, their effect on disease may not be captured comprehensively and can be improved in future versions.
  Not all cytokines with evidence of relevance to RA pathophysiology and treatment are included in the model. Cytokines such as IL-2 and IL-8, have not been explicitly included in the model; IL-2 is assumed to be present in non-limiting amounts in the synovium whereas IL-8’s main role is in the recruitment of PMNs (Poly Morphonuclear Neutrophils). PMNs are not captured in this version of the model since they are not thought to be major drivers of the disease and hence cytokines mainly relevant to PMN activity are also not explicitly captured^39^. Cytokines such as IL-15, IL-18, IL-32 have shown some promise in improving clinical outcomes and may be very relevant to sub-populations of patients but have also not been included at this time. They can be added to the model structure and their functionality can be accounted for explicitly and quantitatively in subsequent versions. RANKL is another cytokine that is not included in the model that may have a major role to play in activating osteoclasts, which are not part of the current physiological scope.
- **Multiple Anti-inflammatory cytokines:** These cytokines down-regulate immune activation and reduce secretion of pro-inflammatory cytokines and are critical feedback to reduce inflammation but are inadequate in RA. We have modeled IL-10 and TGF-β, which are considered to be important anti-inflammatory mediators though, increasing these and other anti-inflammatory cytokines like IL-4 and IL-13 are generally insufficient to reduce disease symptoms in a population^40^.

C. Chemokines/ growth factors
  These are smaller proteins which are critical to immune cell migration to the joint and can also be important therapeutic targets^48^.We have included a general factor regulating recruitment of all leukocytes that can be varied between virtual patients. This factor, called CAMS in the model (for Cell Adhesion Molecules) is at present a function of endothelial cells, to represent the expression of cell adhesion molecules on endothelial cells, and may be expanded further to denote specific adhesion factors^49^. We have modeled RANTES (a key recruiter of T Cells), MIP-3α (recruits T Cells, B Cells and Macrophages), MCP-1 (recruits Macrophages). We also have modeled VEGF, a growth factor that induces the endothelial cells migration (which eventually increases angiogenesis) and exacerbates disease^50^.
D. Serum compartment

Elevated levels of cytokines are observed in both synovium and serum of RA patients. However, the measurements of cytokines in the serum tends to be highly variable and we have not attempted to match observations of serum cytokines to the model^51^. This central compartment in the model serves as a source of immune cells to be recruited into the synovial compartment. Further, the pharmacokinetics of therapy (MTX, ADA and TCZ) are modeled in this compartment and partitioned into the synovial compartment. Currently, we consider the serum compartment only as the input for the PK and as source of immune cells and have not attempted matching immune cell numbers in the serum, which are highly variable.

### Stage 3: Developing Model Structure, Interaction Motifs among Model Components

**Fig. 7:**
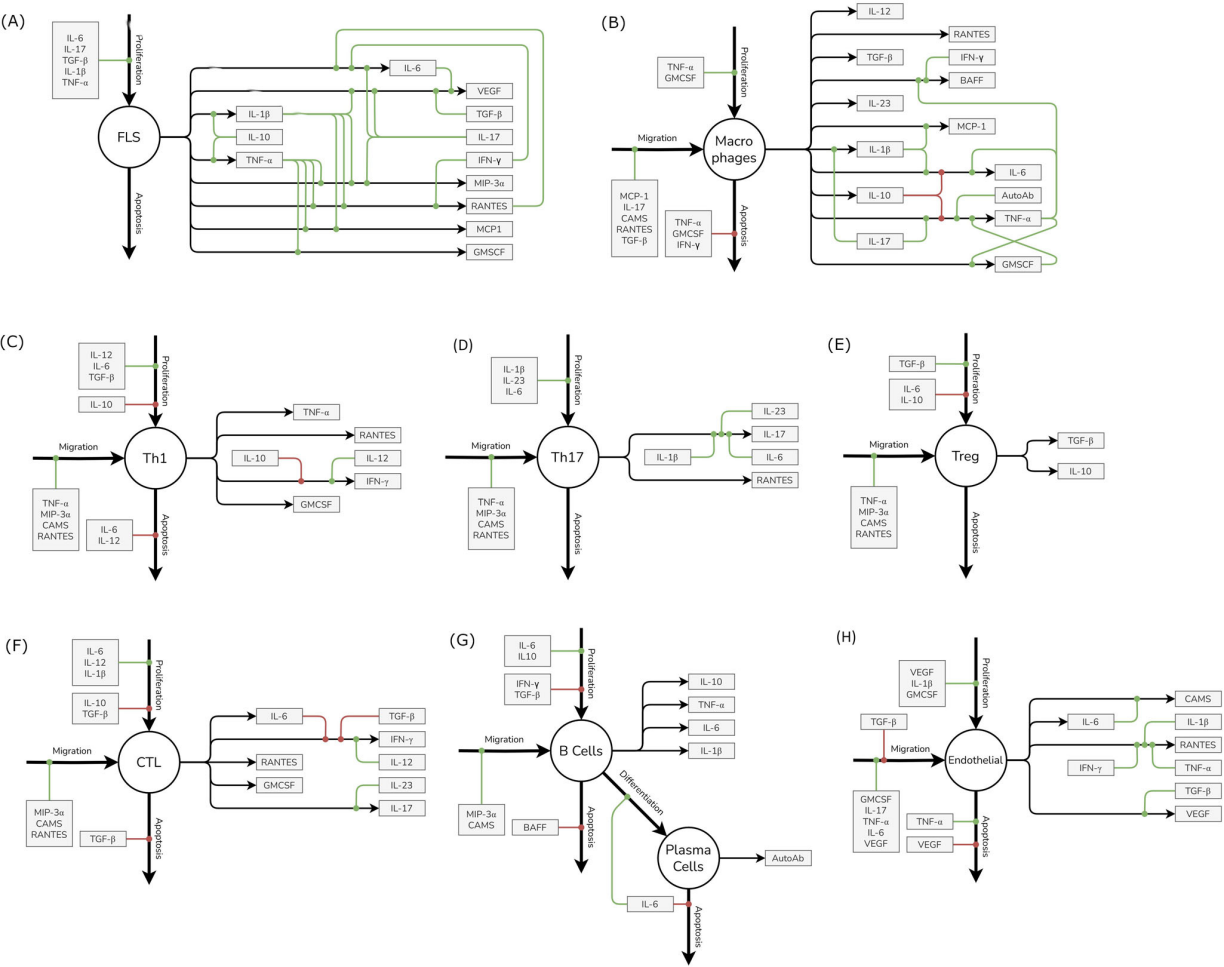
The life cycle and regulation of different cells and cytokines included in the model. The life cycle of each cell type is regulated by three processes - Proliferation, Migration and Apoptosis while it can contribute to secretion of one or more cytokines, chemokines or growth factors. Each of these processes can in turn be regulated by positive and negative regulators which increase or decrease the rates of these processes respectively. Here, the impact of positive regulators is denoted by green lines leading into the respective process while the impact of negative regulators is denoted by red lines. Note that there is no migration of FLS represented in the model; being a structural cell, it is not assumed to migrate via the blood into the synovium (in an analogous manner to the migration of the other cell types in the model). **A** to **H** represent FLS, Macrophages, Th1, Th17, Treg, CTLs, B Cells and Plasma cells and Endothelial cells respectively.

Equations of Cells and Cytokines in the Synovial Compartment: The density of cells and concentration of cytokines is tracked in the synovium as a measure of disease severity (the synovium itself has a fixed volume of 1 mL in all Virtual Patients). Virtual Patients are modeled to have dynamic steady-state concentrations of cells and cytokines in disease which are reduced by therapy. The main motifs of interaction of the various constituents of the synovial compartment are: (i) Inflammatory cells release cytokines (ii) cytokines modulate life cycle of inflammatory and other cells (e.g., rates of proliferation, apoptosis and migration) and (iii) cytokines also modulate rate of secretion of cytokines by the inflammatory cells. The dynamics of each cell type’s density in the synovium is captured in an ordinary differential equation (ODE) and the following life-cycle processes are explicitly captured: proliferation, apoptosis, and migration into the joint from the central compartment (Fig. 1, and Fig. 7). Synovial cytokine concentration is captured by the model equations and tracks secretion of cytokines and other mediators by various cell types as well as their clearance. Each of the cell life-cycle processes are regulated by cytokines as shown in Fig. 1 (with macrophage life cycle as an example). We have assumed that the cells are well-matured, differentiated and activated in our model and these processes are not captured explicitly.

The dynamics of each cell is represented as shown in Eq. 1 below:

1 $$d({Cell})/{dt}={ProlifRate}+{MigRate}-{DegRate}$$

Where ProlifRate, MigRate and DegRate represent the rate of proliferation, migration or influx into the synovium and apoptosis rate of each cell type. Each of these processes is modeled as occurring at a baseline rate (cells/mL/hour) that can be further increased or decreased by cytokines or other mediators. Further, Proliferation and Migration rate are modeled as follows (Eqs. 2 and 3):

2 $${ProlifRate}={BaselineRate}* (1+{ProProlifEffect})* (1-{AntiProlifEffect})$$

3 $${MigRate}={BaselineRate}* (1+{ProMigEffect})* (1-{AntiMigEffect})\left)\right.$$

Where ProProlifEffect and AntiProlifEffect represent the fold increase and decrease in proliferation rate due to the effect of different cytokines that act on the cell. We have modeled the pro-inflammatory and anti-inflammatory effects as independent, with 0 < AntiProlifeffect < 1 and 0 < ProProlifEffect < 10. The effects of the cytokines on cell life-cycle rates are constrained based on available data, which is usually based on in-vitro experiments, and are saturating functions of cytokine concentrations across about 3 log scales. The equations describing these processes and design choices are explained in greater detail in the section ‘Additional information on Methods: 1. Representing cell and cytokine life cycle’.

Several approaches have been used to integrate cytokine signals which can be pleiotropic, redundant, synergistic, or antagonistic and may use overlapping intracellular pathways to affect the signaling^52,53^. These include different ways of combining these signals in differential equation models such as ours as well as logic-based Boolean models^24,54–56^. Our approach is designed to work at the scale of interest of reproducing the clinical effect of perturbations (anti-cytokine therapy) on a clinical population (vs., for example, reproducing cell cultures in an in vitro experiment)^57^. More complex mechanisms such as increasing response of a cytokine by another cytokine (without a direct effect by the first cytokine on the process) or the involvement of obligatory cytokines for an^27^ interaction are not modeled currently.

The dynamics of a cytokine in our model are captured in Eqs. 4–6 below:

4 $$d({Cytokine})/{dt}={CytSecRate}-{CytDegRate}$$

5 $${CytSecRate}=\mathop{\sum }\limits_{i={cell\; types}}^{n}{BaselineRate}* {CellDensit}{y}_{i}* \left(1+{ProSecEffect}\right)* \left(1-{AntiSecEffect}\right)$$

6 $${CytDegRate}={BaselineRate}* {CytokineConc}$$

Where Cytokine is the cytokine concentration, CytSecRate refers to the net cytokine secretion rate (ng/mL/hr) and CytDegRate (ng/mL/hr) refers to degradation rate of the cytokine.

CytDegRate is used from the literature where available, and generally ranges from minutes to hours. Data on cytokine half-life was obtained from multiple sources and often ranges from a few minutes to several hours (see Supplementary Data 2). The value BaselineRate_i refers to the baseline secretion rate by a particular cell type with density CellDensity_i. These rates are estimated to match reported steady-state concentrations of the cytokine. The relative amount secreted by each cell type was estimated to match data when available. ProSecEffect_i is the effect of the positive regulators on the baseline secretion rate and AntiSecEffect_i is the effect of the negative regulators of that particular cell type and are modeled similarly to their effect on cell life cycle rates. Greater detail on assumptions in the modeling are in the section, “Additional information on Methods: 2. Representing cytokine secretion and degradation”).

#### Model Parameterization

In all cases, data is taken from “bottom-up” parameters which determine the rates of the various reactions and is used to directly inform the model parameters. “Top-down” data from clinical, synovial, RA samples is prioritized over clinical sera or pre-clinical measurements to determine model behavior and further constrain the parameter space. Below, we discuss in further detail the sources of parameterization for each subset of model parameters. Documentation of all model parameters estimated from literature and used in the model is available in the Supplementary Data 2.

When possible, ‘bottom-up’ data from in vitro experiments are used to determine the rate constants associated with cellular processes such as apoptosis, migration etc. Mediator driven effects are also determined from in vitro experiments, but the model quantification is limited to ‘reasonable’ fold changes in vivo. For example, the fold increase in proliferation due to cytokines is capped at 10x of baseline rates. When such parameters are unavailable, they are estimated such that the model is consistent with ‘top-down’ data such as measured synovial cell densities and cytokine concentrations at steady-state and in response to therapy (if such measurements are available on therapy). Steady state cell numbers for all cell types used in the model are obtained from literature data on cell numbers in terms of cells/mm^2^ from IHC/ microscopy based examination of biopsy samples of RA patients and converted them into cells/mL based on the method described in Preza et al. ^58^ The experiments and related calculations are described in the “Supplementary Data 1” in the sheet “conversion_ cells_ml”.

In particular, the following are some examples of rate constants of cell life cycle and cytokine secretion used to parameterize the model:

- **Rate of cell apoptosis (1/hour):** This was calculated from the literature data on the % apoptosis seen in activated immune cells (primary cell lines like T Cells, Macrophages etc. derived from healthy human PBMC, usually treated with LPS (vs. passaged cell lines)(See for e.g. ^59^). Among structural cells, for the endothelial cells, half-life measured in inflamed tissue was used^60^. For FLS, the % apoptosis of FLS reported in in-vitro cell cultures of RA -FLS was used^61^. Apoptosis rates for various cell types in in-vitro settings are reported in the literature and tend to range from a few hours to a few days. For e.g., to calculate the half-life of Th1-cells, we used data on activation induced cell death (AICD) i.e., % apoptosis reported in activated Th1 cells when stimulated with anti-CD3^62^. This data showed 15% apoptosis in 6 hours which is then translated into a half-life of 0.65/day using the equation t_1/2_= ln (100/ (100-% apoptosis)/time of measurement in days.
- **Rate of cell migration or influx into synovium (cell/ml/hour):** These rates, when available were determined from migration assays carried out for the different cell types e.g., for determining the baseline (i.e., unregulated) migration rate of CD4 T Cells into the synovium, we looked at an in-vitro migration assay where the fraction of CD4 T cells that cross a semi- permeable membrane in absence of any chemokine in 2 hours was noted^63^. This rate is then increased by different folds in response to different chemokines.

Two different processes contribute to increase in cell density of any cell type in the model - migration or influx of the cell type into the synovium in response to chemokines and proliferation in response to different signals received by the cell type. The relative contribution of synovial cell density due to proliferation within the synovium vs. influx from the serum is poorly characterized in the literature. For e.g., a study tracked autologous labeled macrophages in RA patients and found <0.003% influx into the synovium^64^. No such data was available for lymphocytes. In steady-state disease, in the Vpop, the flux due to proliferation tends to dominate across cell types and Virtual Patients in the model and is consistent with our best understanding based on clinical trial data where anti chemokine therapies have not been successful in RA whereas anti proliferative therapy e.g., Abatacept (Anti-CTLA4) has been successful^65,66^.

- **Rate of proliferation (cell/ml/hour):** Given reasonable constraints on the other 2 fluxes of cell life cycle, rates of proliferation were determined by fitting to obtain the observed steady state number of the cell type in RA. The proliferation rate is assumed to be of zeroth order, as is needed to maintain a steady state of the disease.
- **Rate of cytokine secretion (ng/cell/hour):** For calculating cytokine secretion rate per cell type in the reference Virtual Patient, we have used the following strategy:

- The concentration of cytokine in the tissue (synovial tissue or synovial fluid) is determined from the literature (see the Supplementary Data 2 for references).
- The relative secretion of cytokine by the various cells is determined from literature. For e.g., TNF-α can be secreted by macrophages, Th1 cells, CD8 T cells, B cells and FLS in the model. From literature, we determine that macrophages are the major contributor of the TNF-α seen in the joint, while T-cells are also known to be important secretors of TNF-α^67,68^.
- Cytokine degradation rate is obtained from the reported half-life of each cytokine (see Supplementary Data 2). The cytokine secretion rates were varied during the creation of the virtual cohort and the relative order of secretion rates is not maintained in the final Vpop.

In addition, rates of each of these processes can be regulated by cytokines. Regulation of cell behavior by a cytokine is assumed to be a fold-increase over a baseline rate and is a saturating function of cytokine concentration. In some cases, a particular cytokine may be essential for a behavior e.g., IL-12 is essential for IFN-γ secretion by Th1 cells^69^. In this case, we do not represent the effect as a fold increase, but rather modify the function to make IFN-γ secretion 0 in the absence of IL-12. The maximum effect of a cytokine in increasing or decreasing a rate is capped using a value determined from the literature (see Supplementary Data 2 for the quantification of these values). In the case of multiple cytokines regulating the same effect, the total effect is capped at 10-fold increase or 10-fold decrease from baseline rates (See the section “Additional information on Methods, 3, Representing the effect of multiple cytokines” for the mathematical representation of this). This is a heuristic which we hypothesize avoids unphysiological behavior clinically, though in vitro data may show higher increases. The variability in patient response may be sensitive to this hypothesis and must be investigated further.

Implementation of disease severity scores: Once the basic interactions of the cells and cytokines in the synovium are set up, this physiological scale is connected to clinical disease status. Multiple disease severity scores, such as the DAS28-CRP, ACR, SDAI have been tracked in clinical trials as markers of baseline disease characteristics and response to therapy^70,71^. These measures track subjective and objective assessment of disease severity in RA and do not require measurement of immune cells or mediators from serum or synovium. Our assumption is that the densities of cells driving the disease correlate with disease severity and hence the disease score. This step of connecting the cell densities in the model to disease severity scores, which are based on subjective assessments, while reasonable, is an empirical assumption attached to an otherwise mechanistic model.

Other models have used similar approaches^24^. This connection between disease score and actual disease severity as measured by serum and synovial measurements, imaging of affected joints have been explored with other biomarker studies (Correlation of disease score with RF, anti CCP, serum cytokine levels^71,72^. In particular, a multi-biomarker panel based on serum measurements was developed and validated to (VECTRA^73–75^), other serum proteins. The connection between biomarkers and clinical scores in RA needs further investigation to establish a stronger connection between physiology and disease severity^76–78^.

The model is calibrated to DAS28-CRP and ACR as the clinical scores. DAS28-CRP has been shown to have robust correlations with disease and eventual patient outcomes of quality of life and is being reported in most recent trials^79–81^. Quantitatively, one major advantage is that DAS28-CRP scores are reported at baseline as an entry criterion (vs. the ACR score which is only defined as a change in a study). In developing the expression connecting the synovial read-outs to DAS28-CRP score, using our best understanding of the disease physiology and prominence of different cells to the disease activity, we have allocated different coefficients to different cell types to contribute to the final score. The other parameters such as Kms and γs were calibrated based on the physiological steady state ranges for their corresponding cell types.

7 $$\begin{array}{l}{DAS}28-{CR}{P}_{{model\; score}}=2.5* {Hill}({Macrophages})+2* {Hill}({FLS})+1.5* {Hill}({Th}1)\\\qquad\qquad\qquad\qquad\qquad\quad\;\; +\,1.5* {Hill}({BCell})+1* {Hill}({PlasmaCell})+0.5* {Hill}({Th}17)\\\qquad\qquad\qquad\qquad\qquad\quad\;\;+\,0.5* {Hill}({Endothelial}) +0.5* {Hill}({CTL})-0.5* ({Treg})\end{array}$$

Where

$${Hill}({Cell}{\rm{\_}}x)=\frac{{{Cell}{\rm{\_}}x}^{{gamma}{\rm{\_}}x}}{{{Cell}{\rm{\_}}x}^{{gamma}{\rm{\_}}x}+{{Km}{\rm{\_}}x}^{{gamma}{\rm{\_}}x}}$$

The values of Km and γs for different cell types are provided in Table 2 along with the methodology using which they were calibrated (See ‘Additional information on Methods: 4. Representing DAS28-CRP). As mentioned earlier, the ACR score does not have a baseline and is tracked as a change from baseline. For this reason, we have calculated the ACR score in our model as a percent change in the DAS28-CRP, from baseline. We made this as the simplest assumption given limited understanding of the physiological differences between these scores.

The DAS28-CRP in the model is driven by the best available constraints and modeler choices to maintain a simple, transparent structure. Other modelers may be motivated to derive a more intricate mathematical model to connect synovial species and clinical scores, and this can be a major area of model refinement if more data is available in proprietary or other data sources.

### Stages 4 and 5: capturing behaviors, building confidence and exploring variability

#### Characterizing steady-state behavior

**Fig. 8:**
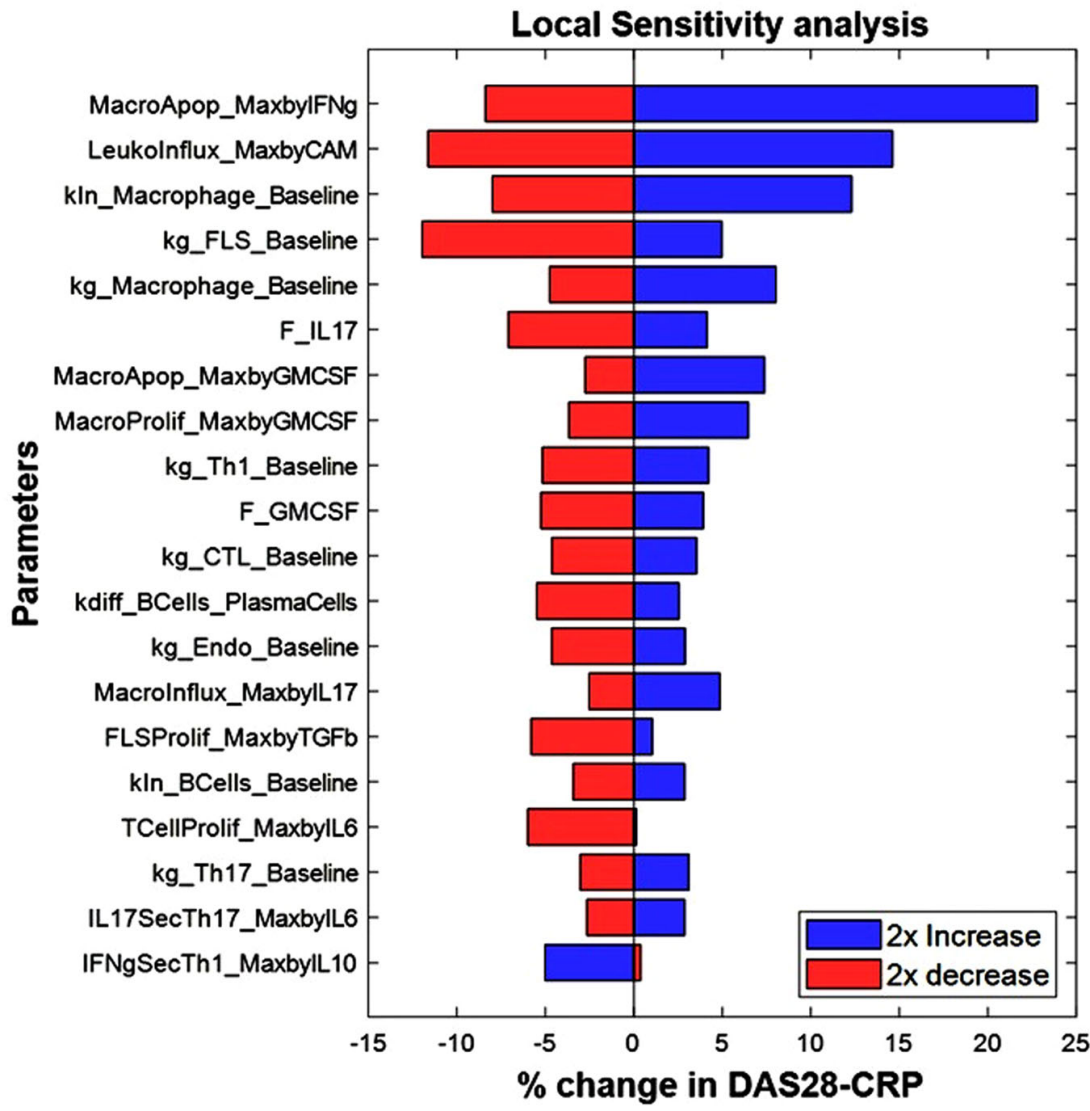
Local sensitivity analysis of a representative Virtual patient from the Vpop, showing the top 20 most sensitive parameters in this patient. The figure shows the percent change in DAS28-CRP score observed when introducing 2x variability to the parameter values of the virtual patient with the blue bars representing the outcomes of a 2x increase and the red bars representing the outcomes of a 2x decrease in the value of the parameter. E.g., on increasing the parameter MacroApop_MaxbyIFng from its baseline value by 2x, there is a 25% increase in the DAS28-CRP score while a 2x reduction in the same parameter leads to an 8% reduction in the DAS28-CRP score.

**Fig. 9:**
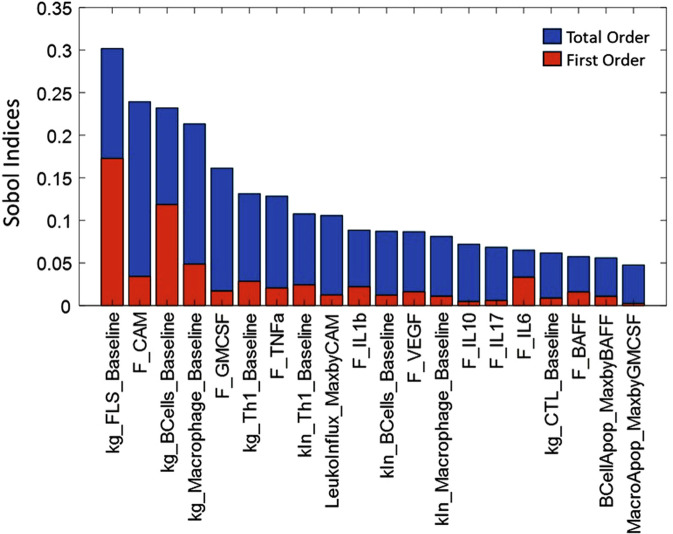
Global sensitivity analysis of the model showing the top 20 most sensitive parameters, showing the total order and first order sensitivities of these parameters. The total order (represented by the blue bars) indicates the contribution of a particular parameter to total variance in the outcome; including its impact due to joint parameter variations while the first order sensitivity (represented by the red bars) indicates sensitivity to the particular parameter alone. E.g., we can see that kg_FLS_Baseline contributes maximally to the total variation seen in the DAS-28CRP score, both directly and through its interactions with other parameters.

Cell numbers were derived from synovial biopsies of RA patients with established disease of moderate to severe severity while cytokine values were derived either from synovial biopsies or synovial fluid samples. We derive the ranges for each cell type/cytokine from the data reported in either synovium or synovial fluid (see Supplementary Data 1). Further we have performed Local and Global Sensitivity Analyses and have identified key model parameters that influence the disease state as shown in Figs. 8 and 9 (Also see ‘Additional information on methods: 5. Sensitivity Analysis of the model). We ensured that all the virtual patients used in Vpop calibration are constrained to lie within the range derived from literature.

A reference virtual patient (ref VP) was generated by calibrating the following parameters to replicate the median cell and cytokine behavior.

1. Proliferation of Cells
2. Secretion rate of cytokines

Once the reference virtual patient was calibrated, we generated multiple virtual patients, i.e., a virtual cohort, (n ~ 200000) by varying the following parameters.

1. Proliferation and migration rates of cells
2. Secretion rate of cytokines
3. Upregulation/Downregulation of cytokines on cell processes

The cell ranges from literature are used as constraints on the generated virtual patients to determine which patients are ‘physiologically plausible’ and will be included during calibration to therapies. This set of plausible virtual patients (n = ~50000) is termed as a Virtual Cohort. While developing this virtual cohort, we have given greater importance to the values of cells than to the values of cytokines determined from the literature. This is because cytokine measurements are generally not from the tissue itself (some are from synovial fluid, while others are from plasma) whereas cell measurements are from the inflamed tissue. It is at this stage that we additionally constrain the virtual patients such that their DAS28-CRP is >3.2.

#### Characterizing response to therapy

After virtual cohort generation to ensure reasonable behavior of virtual patients at steady-state i.e., in absence of therapy, we calibrated the response of the virtual patients to different therapies via Vpop generation. The different criteria based on which we selected therapies for calibration are discussed in the section below (Selection criteria for trials for model calibration). In some cases, a particular cytokine may be essential for a behavior e.g., IL-12 is essential for IFN-γ secretion by Th1 cells^69^. In this case, we do not represent the effect as a fold increase, but rather modify the function to make IFN-γ secretion 0 in the absence of IL-12. Based on these criteria, we selected Methotrexate (MTX), Anti-TNF-α and Anti-IL-6R as the drugs to which the response of the Vpop is matched. We selected MTX therapy to model since this is considered as an ‘anchor drug’ for RA^79,80^ and most patients entering clinical trials for other therapies display an inadequate response to MTX or continue to be on MTX therapy to control RA symptoms^82,83^. When patients show an inadequate response to MTX, biologic therapy is often the next line of treatment. Anti-TNF-α is the first biologic prescribed to RA patients who have inadequate response to DMARDS such as MTX. Five Anti-TNF-α have been approved for RA so far - Infliximab, Etanercept, Adalimumab, Certolizumab Pegol and Golimumab^84^. All of them have similar efficacy in terms of remission and response but have different modes of delivery (Infliximab is an IV drug while the others are given SC) and different half-lives^85^. Of these, Adalimumab was selected for model calibration. Tocilizumab or Anti IL-6R was included into the model calibration since it is one of the therapies (along with Rituximab or Abatacept) that are tried out in anti-TNF-α IR patients^84^. The model also has adequate structure to implement novel therapies like Anti-IL-17, Anti-IL-1 and JAK/Tyk inhibitors etc. that are developed to address non-responders to the first line therapies.

Selection criteria for trials for model calibration: The design and purpose of the model needs to be considered carefully in selecting the trials to calibrate the Vpop against. The clinical trials for the therapies to be calibrated are selected using the following criteria:

- Steady-state, induction phase trials: In this case, the model developed is of steady-state disease (for < 12 months) and does not consider disease progression and therefore induction trials are selected for model calibration. Maintenance phase trials are not considered in this model since these trials are for longer duration (>12 months) and will need to include disease progression which is not a feature of this model.
- Trial design: Trials with placebo arms are prioritized; in absence of any trials with a placebo arm, a trial with a common comparator of already existing therapy can be used. Trial protocols should be carefully examined for any changes such as crossover design which can complicate the interpretation of results. Phase III trials are generally preferred because of the large size of the sample.
- Patient population: Different population characteristics are of interest to the community and should be considered when calibrating the Vpop. Anti-TNF-α therapy is the recommended first biologic therapy for inadequate responders to DMARDS. Hence, anti-TNF-α non-responders and anti-TNF-α naive patients are explicitly recruited, in RA clinical trials for novel therapies to determine the response of both types of patients to novel therapies (e.g., see^17^. The Vpop generated from the model should contain both these sub-populations.
- Trial outcomes: It should be noted that clinical trials of interest should have the clinical readout of interest. For example, if ACR and DAS28-CRP are not reported in a clinical trial paper, it may not be preferred. For example, in the context of RA, the distribution of DAS28-CRP scores at baseline, change in DAS28-CRP mean value at different time points at different doses, remission and response scores should be recorded and investigated for conflict or concord.

Treatment of Placebo: The gold standard for judging the efficacy of a novel therapeutic is the randomized, controlled, clinical trial where the remission and response seen in the treatment arm should be compared to that seen in the control arm^86^. Patients with autoimmune disease can show spontaneous response/remission, hence, a quantitative comparison of therapy with placebo as the comparator can be useful to determine efficacy. However, placebo correction by absolute subtraction of placebo arm remission from treatment arm remission does not account for the possibility that patients who respond to placebo can also respond to therapy. This can lead to an underestimation of the true therapeutic efficacy of the drug. To account for this, Wang et al. ^19^ uses a method of probabilities to arrive at a placebo correction method, which we have used in our model^19^.

The summary of trial characteristics of selected trials for model calibration are shown in Table 1.

**Table 1:** Top-down data on baseline characteristics and response to MTX, ADA, TCZ as reported in the selected Clinical trials

	Baseline characteristics				After Therapy					
Trial/Therapy of interest	Arms	N	Previously took anti-TNFs (in %)	DAS28-CRP	ACR 20	ACR 50	ACR 70	% DAS28-CRP < 2.6	%DAS 28-CRP < 3.2	% Δ DAS28-CRP > -1.2
Methotrexate (MTX) (19)	MTX	182	0	NR	46	23	9	NR	NR	NR
	Placebo	118	0	NR	26	8	4	NR	NR	NR
Adalimumab (ADA) OPTIMA trial (17)	ADA (40 mg Q2W) + MTX	515	0	6.0 ± 1.0	70	52	35	34	47	NR
	Placebo+ MTX	517	0	6.0 ± 1.0	57	34	17	17	26	NR
Tocilizumab (TCZ) – ROSE trial (18)	TCZ 8 mg/kg + DMARDS	409	37.9	6.53 ± 1.03	45	30.1	13.9	38.4	50.7	87.9
	Placebo +DMARDS	205	38	6.55 ± 1.01	25	11.2	1.9	2	10.4	53.4

NR= Not Reported. DAS28-CRP scores (Remission is defined as DAS28-CRP < 2.6, while response is defined as DAS28-CRP < 3.6 and delta DAS28-CRP > 1.2) as reported in the clinical trials.

It is to be noted that the trials for Adalimumab and Tocilizumab were run in combination with Methotrexate and with Placebo + Methotrexate as the control arm. Due to this, we have considered the placebo corrected clinical outcomes to represent the efficacy of Adalimumab and Tocilizumab monotherapies and used for the calibration of monotherapies in the model.

#### Modeling PK and PD of the therapies

The model captures the PK of the drug in the tissue and serum levels using the simple two or three compartment models. The PD of the drug is captured by using a binding kinetics model relevant to the drug.

1. PK modeling: The parameters related to PK of the therapies in the model were taken from literature and are described in the section, “Additional information on Methods, 6. Pharmacokinetics and Pharmacodynamics of therapies”.
2. PD modeling:

- Anti-cytokine therapies such as Adalimumab are modeled using the in-vitro binding parameters assuming equilibrium kinetics^87,88^.
- Anti-Cytokine Receptor therapy such as Tocilizumab is modeled with an assumption that the reduction in the free receptor has equivalent effect as similar fold-reduction in free cytokine concentration. This assumption is made because the cytokine functions by binding to its receptor and hence blocking the receptor should have equivalent effects as blocking the cytokine itself.
- MTX therapy affects (a) cytokine secretion (b) migration and (c) Treg function^89–92^. The effect of the dose-response in each pathway is adjusted to match the clinical readout. The equations representing the PD are in the section, “Additional information on Methods, 6. Pharmacokinetics and Pharmacodynamics of therapies”.

#### Development of Virtual Population

**Fig. 10:**
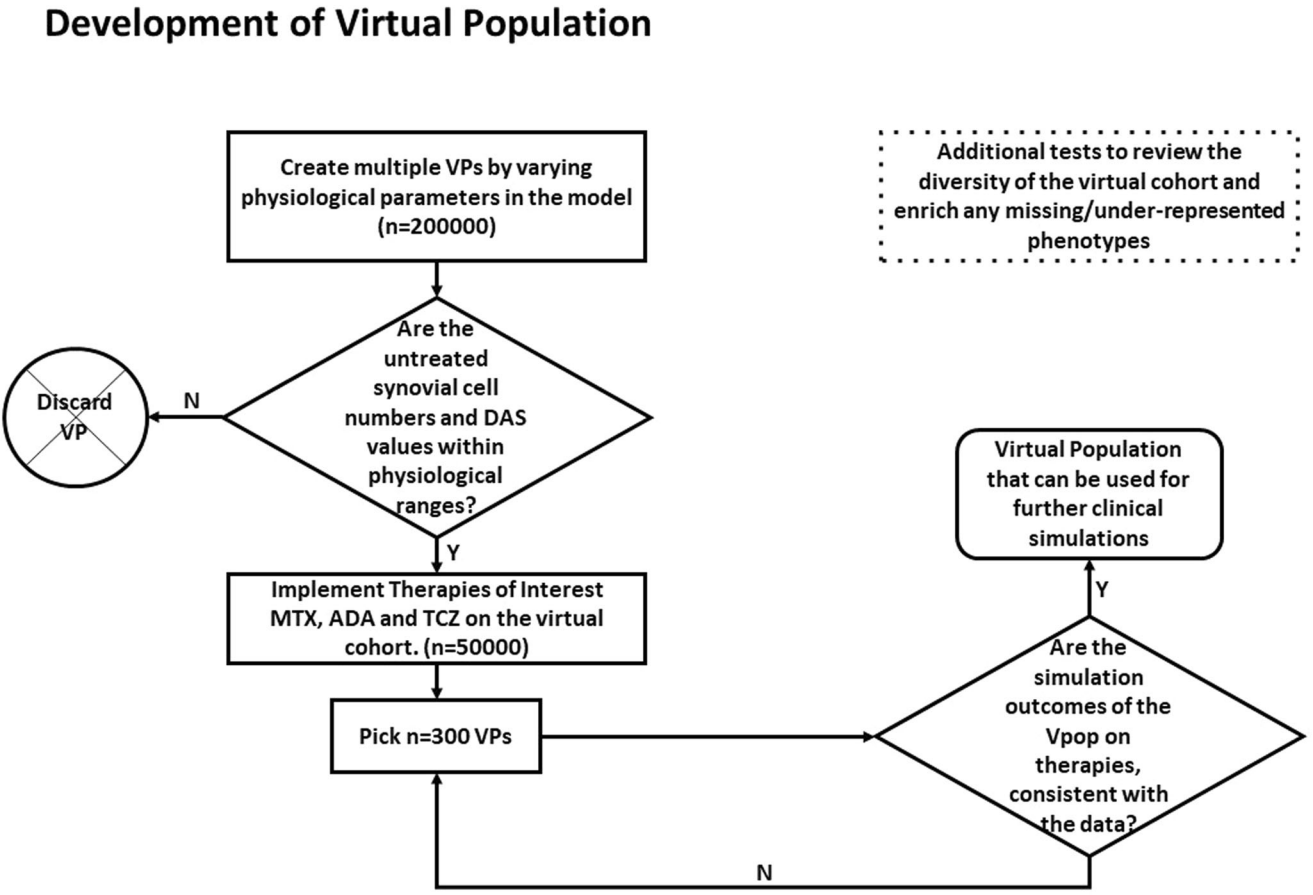
The process of creation and calibration of a Vpop from a virtual cohort. Flow chart depicting the process of creation of a virtual cohort and its calibration to multiple therapies to generate a Virtual population.

The selected patients were then subjected to the therapies to which the Vpop is to be calibrated (here, MTX, Ada and TCZ). A subset of the virtual cohort, which shows appropriate baseline characteristics and responses to therapies - as determined by comparison with the values noted from the clinical trials, is selected to form the final Vpop. A Vpop of ~300 VPs were selected using this method. This process is described in Fig. 10. The distribution plots for the model parameters in the Vpop and the distribution plots of resultant steady state cell and cytokine concentrations are included in the section, “Additional information on Methods, 7. Development and characterization of Vpop”.

Cohort Enrichment: We had to enrich a few patient phenotypes in the cohort before we were able to generate a reasonable Vpop. Enrichment involves recognizing the under-represented phenotypes in the cohort that need to be more prevalent to obtain a good Vpop and generating similar plausible virtual patients by re-sampling from a tighter parameter space around them^93^. This process can be iterative. To make the cohort enrichment efficient, we used prevalence weighted Vpop generation using gQSPsim and used the patients with very high prevalence weights to be enriched^94^. Other groups have developed QSP models with multiple Vpops in order to improve the coverage of the plausible parameter space and offer even more robust predictions^26,95^. Developing multiple VPops for this model is definitely a future area of improvement.

### Additional Information on methods

1. Representing Cell and cytokine Life cycle: The life cycle and regulation of the cells and cytokines represented in the model are shown in Fig. 7. The species in the model can be broadly classified into cells and soluble mediators. Each of these undergoes distinct processes. Cells undergo the following reactions: Proliferation, Influx and Degradation. The ODE for a cell type is written as follows:

8 $$d({Cell})/{dt}={ProlifRate}+{InfRate}-{DegRate}$$

Calculation of rates of proliferation, influx and degradation of the cell are described below.

- Cell proliferation rate is calculated as:
  9 $${ProlifRate}={BaselineRate}* \left(1+{ProProlifEffect}\right)* (1-{AntiProlifEffect})$$
  where
  **ProlifRate** is the rate of proliferation rate of each cell type, expressed in cells/(milliliter*day)
  **BaselineRate** is the baseline rate of proliferation, in absence of all modeled cytokines, expressed in cells/(milliliter*day)
  **ProProlifEffect** is the increase in the proliferation rate due to all cytokines which cause an increase in proliferation of that cell type.
  **AntiProlifEffect** is the reduction in the proliferation rate due to all cytokines which cause a decrease in proliferation of that cell type.
  We have chosen to model proliferation rate as a zeroth order term as it may be a more accurate representation of growth in a crowded, growth restricted environment, as well as allows the model to settle into a stable steady-state balancing the zeroth order proliferation and first order clearance. The combination of multiple cytokines is discussed in the next section and in the next section.
- Cell migration or influx rate is calculated as:
  10 $${InfRate}={BaselineRate}* \left(1+{ProInfEffect}\right)* \left(1-{AntiInfEffect}\right)* (1-{AntiInfMTX})$$
  where
  **InfRate** is the rate of influx rate of each cell type, expressed in cells/(milliliter*day)
  **BaselineRate** is the baseline rate of influx expected in absence of all modeled cytokines, expressed in cells/(milliliter*day)
  **ProInfEffect** is the proportional increase in the influx rate because of the cytokines/chemokines which enhance influx of each cell type.
  **AntiInfEffect** is the proportional reduction in the influx rate because of the cytokines/chemokines which reduce influx of each cell type.
- Cell degradation rate is calculated as:

11 $${DegRate}={BaselineRate}* {CellDensity}* \left(1+{ProDegEffect}\right)* \left(1-{AntiDegEffect}\right)$$

where

**DegRate** is the rate of removal of the cell type, expressed in cells/(milliliter*day)

**BaselineRate** is the baseline rate of degradation of the cell type expected in absence of all modeled cytokines, expressed in 1/day

**CellDensity** is the density of the cell type expressed in cells/milliliter

**ProDegEffect** is the proportional increase in the degradation rate of the cell type because of the cytokines which cause an increase in degradation of each cell type.

**AntiDegEffect** is the proportional reduction in the degradation rate of the cell type because of the cytokines which cause a decrease in degradation of each cell type.

2. Representing Cytokine secretion and degradation: Cytokines undergo the following reactions: Secretion and Clearance. The ODE for a cytokine is the sum of these reactions as follows:
  12 $$d({Cytokine})/{dt}={CytSecRate}-{CytDegRate}$$
  The secretion rate for each cytokine is a sum of secretion rates by several cell types and has the structure:
  13 $$\begin{array}{l}{CytSecRate}=\mathop{\sum }\limits_{i}\left({BaselineRat}{e}_{i}* {CellDensit}{y}_{i}* (1+{ProSecEffec}{t}_{i})\right.\\\qquad\qquad\qquad\;\;* \left.(1-{AntiSecEffec}{t}_{i})\right)\end{array}$$
  where
  **CytSecRate** is the total rate of secretion of the cytokine, expressed in nanogram/(milliliter*day)
  **BaselineRate** is the baseline rate of secretion of the cytokine by a cell type expected in absence of all modeled cytokines, expressed in nanogram/(cell*day)
  **CellDensity** is the density of each cell type expressed in cell/milliliter
  **ProSecEffect** is the proportional increase in the secretion rate of the cytokine by a cell type because of the effect of other cytokines on the cell.
  **AntiSecEffect** is the proportional reduction in the secretion rate of the cytokine by a cell type because of the effect of other cytokines on the cell.
  The clearance rate of a cytokine is expressed as follows:
  14 $${CytDegRate}={BaselineRate}* {CytokineConc}$$
  where
  **CytDegRate** is the rate of removal of the cytokine, expressed in nanogram/(milliliter*day)
  **BaselineRate** is the baseline rate of clearance of the cytokine expressed in 1/day
  **CytokineConc** is the concentration of the cytokine expressed in nanogram/milliliter
3. Representing effect of multiple cytokines: As observed above, the rates of reactions have been moderated using terms such as:
  $$\left(1+{ProRate}\right)\,{\rm{and}}\,\left(1-{AntiRate}\right)$$
  These refer to the increase and decrease in the cell life-cycle rates because of the complex cytokine milieu of the synovium. Each term is a sum of individual contributions from several cytokines and has the following structure:
  15 $${ProRate}=\min ({Prolimit},\sum _{{cytokines}}{MM}({CytokineConc},{VmEffect},{KmEffect},{SlopeEffect}))$$
  16 $${AntiRate}=\min ({Antilimit},\sum _{{cytokines}}{MM}({CytokineConc},{VmEffect},{KmEffect},{SlopeEffect}))$$
  where
  **ProRate** is the proportional increase in any rate because of the surrounding cytokines
  **Prolimit** is the limit imposed on the proportional increase of any rate to accommodate biological feasibility; currently, this is fixed at 10-fold.
  **Antilimit** is the limit imposed on the proportional decrease of any rate to accommodate biological feasibility; currently this is fixed at 0.75.
  **MM** is an external function that calculates the value of the Hill expression
  $$\frac{{VmEffect}* {{CytokineConc}}^{{SlopeEffect}}}{{{CytokineConc}}^{{SlopeEffect}}+{{KmEffect}}^{{SlopeEffect}}}$$
  **VmEffect** is the maximum possible increase in a rate by the cytokine
  **KmEffect** is the concentration of the cytokine concentration at which the rate is increased by half the maximum possible increase by the cytokine
  **SlopeEffect** is the hill coefficient of the hill function
  **CytokineConc** is the concentration of the cytokine expressed in nanogram/milliliter
  Both Pro and Anti effects are capped to lead to a maximum 10-fold change in the activity. But they hold different values by nature of how they are used in the equations. For example, ProProlifeffect is used as “Rate = Baseline*(1+ProProlifeffect)” and a cap of 10 on ProProlifEffect leads to a maximum of “Rate = 11*Baseline”. Meanwhile, AntiProlifeffect is used as “Rate = Baseline(1-AntiProlifeffect)” and a cap of 0.9 on AntiProlifeffect leads to a minimum of “Rate = 0.1*Baseline”. Both lead to a similar fold change from baseline, but in opposite directions.
4. Representing DAS28-CRP score: The following expression is used to calculate the DAS28-CRP value of the virtual patients.
  17 $${DAS}28-{CR}{P}_{{model\; score}}=2.5* {Hill}({Macrophages})+2* {Hill}({FLS})+1.5* {Hill}({Th}1)+1.5* {Hill}({BCell})+1* {Hill}({PlasmaCell})+0.5* {Hill}({Th}17)+0.5* {Hill}({Endothelial})+0.5* {Hill}({CTL})-0.5* ({Treg})$$
  Where
  $${Hill}({Cell}{\rm{\_}}x)=({Cell}{{Density}{\rm{\_}}x}^{{\rm{\wedge }}}{gamma}{\rm{\_}}x)/({{Cell}{\rm{\_}}{Density}{\rm{\_}}x}^{{\rm{\wedge }}}{gamma}{\rm{\_}}x+{{Km}{\rm{\_}}x}^{{\rm{\wedge }}}{gamma}{\rm{\_}}x)$$
  The values of Km and gamma for each cell type are in Table 2 below.

**Table 2:** The table shows the parametrization for the DAS28-CRP calculation in the model, estimated to ensure a uniform distribution of the disease score between 0 to 10 across the cohort

Cell Type	Km	gamma
FLS	1.3E7	2.5
Endothelial	4.2E7	2.5
Macrophages	2.2E7	2.5
Th1	4.0E6	2.5
Th17	1.0E5	2.5
Tregs	2.0E6	2.5
B-Cells	3.0E6	2.5
Plasma Cells	1.8E6	2.5
  The Km_x for each cell_x is calibrated such there is a uniform distribution of ${Hill}({Cell\_x})$ from 0 to 1, at baseline for the cohort and that eventually, overall DAS28-CRP is well distributed between 0 and 10. The gamma_x was also chosen to be at 2.5, to achieve the mentioned behavior. The gamma_x is fixed to be the same across all cell types such that similar fold reductions in the cell densities translate to similar reductions in their subscores. Once the DAS28-CRP score has been calibrated, Virtual Patients in the cohort with baseline DAS28-CRP < = 3.2 were removed from the cohort as they correspond to low disease activity and are not eligible for the clinical trials.
5. Sensitivity Analysis of the Model: We carried out both local and global sensitivity analyses on the parameters of the model to understand the dependence of the model outcome on the different parameters of the model.
  - Local Sensitivity Analysis: From the VPop, the virtual patient closest to the median synovial cell densities was picked for this analysis. To measure the sensitivity of each parameter, the percent change in DAS28-CRP score is observed by introducing 2x variability to the parameter value of the virtual patient. The top 20 sensitive parameters are plotted as a tornado plot as shown in Fig. 8. As can be seen from the figure, macrophage related parameters are the most sensitive ones in this patient, along with parameters affecting influx of all immune cells (Leukoinflux_MaxbyCAM), FLS proliferation (kg_FLS_baseline), Th1 proliferation etc. This is in line with our expectations that macrophages are important drivers of the disease. Note that these sensitivities are likely to be different in other patients in the Vpop; in fact, this is desirable since we want a diversity of responses to therapy, driven by a diversity of underlying mechanisms of disease. The limitation of the local sensitivity analysis is that the sensitivity observed to the parameters may be very specific to the virtual patient/parametrization around which the variability is generated. This means that the local sensitivity analysis may not represent the sensitivity of the model across the whole parameter space explored.
  - Global Sensitivity Analysis: To address the shortcomings of the local sensitivity analysis, we have also run a global sensitivity analysis. The first order and total order Sobol indices have been calculated for each parameter using a sample size of 10000 within the explored parameter space. The total order indicates the contribution of a particular parameter to total variance in the outcome; including its impact due to joint parameter variations while the first order sensitivity indicates sensitivity to the particular parameter alone^96^. The top 20 sensitive parameters have been plotted in Fig. 9. As can be seen from the figure, FLS, macrophage and B cell related parameters are the ones which show the most impact on disease severity; the parameter F-CAM regulates the influx of all cell types in the model and is a lumped representation of multiple chemokines. Total order is greater than first order sensitivity for most of the parameters, indicating that co-variance/interactions between the parameters contributes more to model variability than variability in each single parameter.
6. Pharmacokinetics and Pharmacodynamics of therapies: The pharmacokinetics of the drugs used are modeled and parameterized based on the existing work in public literature. The PK models of Methotrexate, Adalimumab and Tocilizumab are implemented as described as summarized in Table 3.

**Table 3:** The details of PK models used for Methotrexate, Adalimumab and Tocilizumab and corresponding references

	Model	Reference Publication
Methotrexate	2-compartmental model with bolus dosing	^97^
Adalimumab	1-compartmental model with SC dosing	^98^
Tocilizumab	2 compartmental model with IV dosing	^99^

- Methotrexate PD: Methotrexate is modeled to affect the system in the model, in the following three routes^89–92^.

1. Reduce the secretion rate of cytokines by all cells except Tregs.
2. Increase the production rate of Anti-Inflammatory cytokines by Tregs.
3. Reduce the influx rate of Immune cells into the synovium.
  These effects are calculated using the following Hill expressions, respectively:
  18 $$\begin{array}{ll}{Ant}{i}_{{CytSe}{c}_{{MTX}}}={MM}\left({MTXDrug}{{\_}}{Central}{{\_}}{available},{Anti}{{\_}}{CytSec}{{\_}}{MaxbyMTX},\right.\\\left.\qquad\qquad\qquad\quad{HalfEffectConc}{{\_}}{Anti}{{\_}}{CytSec}{{\_}}{byMTX},{Slope}{{\_}}{Anti}{{\_}}{CytSec}{{\_}}{byMTX}\right)\end{array}$$
  19 $$\begin{array}{ll}{Pro}{{\_}}{CytSec}{{\_}}{MTX}={MM}\left({MTXDrug}{{\_}}{Central}{{\_}}{available},{Pro}{{\_}}{CytSec}{{\_}}{MaxbyMTX},\right.\\\left.\qquad\qquad\qquad\qquad\qquad{HalfEffectConc}{{\_}}{Pro}{{\_}}{CytSec}{{\_}}{byMTX},{Slope}{{\_}}{Pro}{{\_}}{CytSec}{{\_}}{byMTX}\right)\end{array}$$
  20 $$\begin{array}{ll}{Pro}{{\_}}{CytSec}{{\_}}{MTX}={MM}\left({MTXDrug}{{\_}}{Central}{{\_}}{available},{Anti}{{\_}}{CellInflux}{{\_}}{MaxbyMTX},\right.\\\left.\qquad\qquad\qquad\qquad\qquad{HalfEffectConc}{{\_}}{Anti}{{\_}}{CellInflux}{{\_}}{byMTX},{Slope}{{\_}}{Anti}{{\_}}{CellInflux}{{\_}}{byMTX}\right)\end{array}$$
  Where
  ${MM}({DrugConc},{VmEffect},{KmEffect},{SlopeEffect})$ is an external function that calculates the value of the Hill expression
  $$\frac{{VmEffect}* {{DrugConc}}^{{SlopeEffect}}}{{{DrugConc}}^{{SlopeEffect}}+{{KmEffect}}^{{SlopeEffect}}}$$
  ${VmEffect}$ is the maximum possible proportional change in a rate by the drug.
  ${KmEffect}$ is the concentration of the cytokine concentration at which the rate is changed by half the maximum possible change by the drug.
  ${SlopeEffect}$ is the hill coefficient of the hill function.
  *Vm*, *Km* and *SlopeEffect* are calibrated at 0.5, 1E-5 mg/L and 2, respectively, to achieve a reasonable mean reduction in disease severity score with therapy.
  ${MTXDrug\_Central\_available}$, expressed in milligram/liter, is the central concentration of methotrexate multiplied by the bioavailability of the drug.
  ${Anti\_CytSec\_MTX}$ is the proportional reduction in the secretion of the proinflammatory cytokines
  ${Pro\_CytSec\_MTX}$ is the proportional increase in the secretion of the anti-inflammatory cytokines
  ${Anti\_CellInflux\_MTX}$ is the proportional reduction in the influx of the immune cells.
  The therapy effect is achieved by multiplying the corresponding cytokine secretion rates by pro-inflammatory cells, cytokine secretion rates by Tregs and the Influx rates of cells into synovium by $(1-{Anti\_CytSec\_MTX})$, (1 + ${Pro\_CytSec\_MTX})$ and $(1-{Anti\_CellInflux\_MTX})$, respectively.
  - Adalimumab PD: The effect of adalimumab is modeled as a reduction in the free TNF-α, due to the formation of an ADA_TNF- α complex, after binding to the drug in synovium.
  - Tocilizumab PD: Tocilizumab targets the IL-6 receptor. As there is no explicit representation of IL-6 receptor in the model, we have assumed that a similar PD effect can be achieved by reducing the IL-6 by a similar proportion as the receptor is expected to be. This is implemented by dividing the clearance of IL-6 with ${KD\_TCZ}/({TCZDrug\_Synovium}+{KD\_TCZ}).$

The above factor corresponds to the fold reduction expected in IL-6 receptors, in a scenario where the Drug concentration is significantly higher than the receptor IL-6 concentration.

**Fig. 11:**
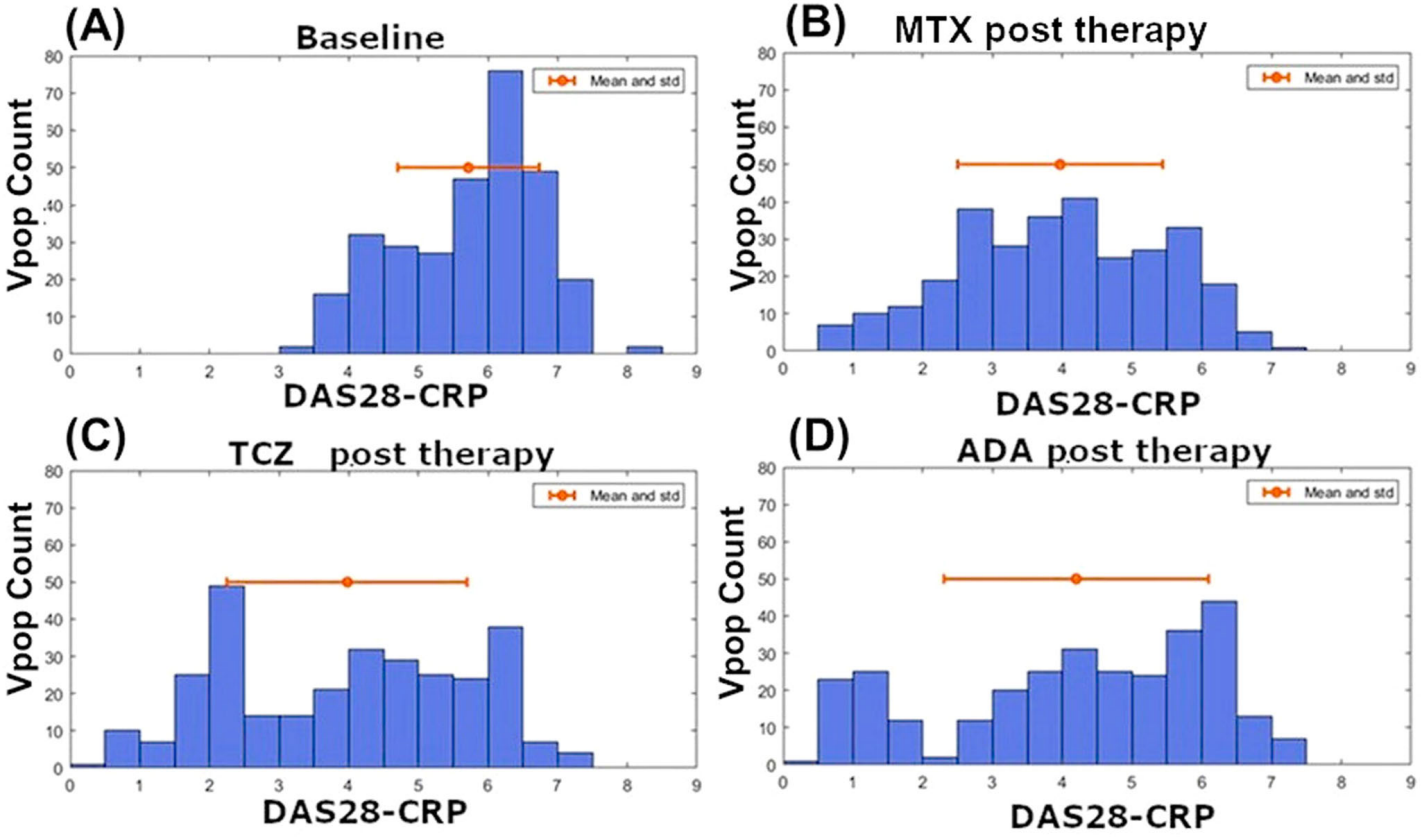
Distribution of DAS-28 score in the Vpop. The DAS28-CRP score distribution in the Vpop (**A**) at baseline, (**B**) post MTX, (**C**) post Adalimumab and (**D**) post Tocilizumab therapies. The red dot indicates the mean while the red line indicates standard deviation. The figures show the distribution of DAS28-CRP scores before and after each therapy. The average baseline DAS28-CRP scores were matched to the mean and standard deviation reported in clinical trials (See Supplementary Data 1). Post therapy DAS28-CRP scores indicate that there is a broad distribution of responses seen in the simulations; indicative of phenotypic diversity in the Vpop.
  - Variability was introduced to a subset of the model parameters to generate the virtual population. Figure 12 describes the distributions of these parameters in the virtual population on a logarithmic X-axis, while Table 4 shows the ranges of the parameters shown in Fig 12 and provides a key to read Fig 12.

7. Development and characterization of the Vpop:
  - The Vpop was generated using a standardized workflow depicted in Fig. 10.
  - *The distribution of clinical scores before and after therapy was determined as shown in* Fig. 11*to confirm that the Vpop is not biased and has a broad distribution of clinical scores*.

**Fig. 12:**
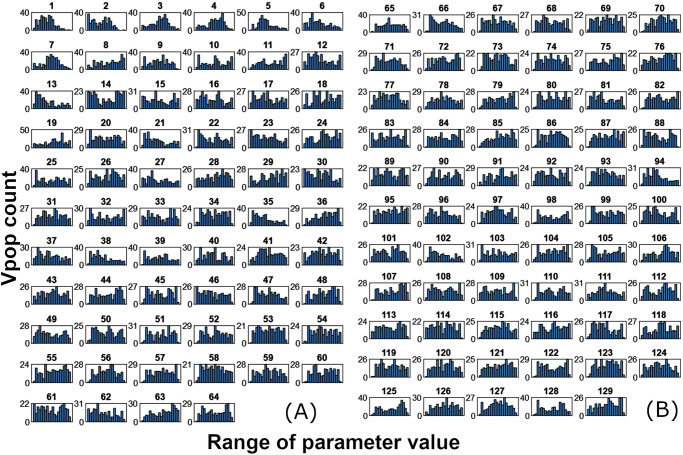
Distribution plots for the parameters varied to generate the Vpop. **A** Distribution plots for the parameters varied to generate the Vpop (1-64) (**B**) Distribution plots for the parameters varied to generate the virtual population (65-129). The parameter distribution shows a broad range, indicating that there is sufficient phenotypic diversity in the Vpop. See Table 4 for the key to read these plots and the bounds for the distribution plot corresponding to each parameter.

**Table 4:** The parameters varied for the virtual population and the corresponding ID numbers in Fig. 12

ID	Parameter	LB	UB	ID	Parameter	LB	UB
1	kg_BCells_Baseline	9.55E + 02	6.61E + 07	66	MacroInflux_MaxbyMCP1	2.30E-01	1.20E + 01
2	kg_CTL_Baseline	1.26E + 03	3.16E + 07	67	IL6SecFLS_MaxbyIFNg	1.41E-01	8.53E + 00
3	kg_Endo_Baseline	4.62E + 05	2.07E + 07	68	IL1bSecFLS_MaxbyIL10	6.30E-02	3.81E + 00
4	kg_FLS_Baseline	1.55E + 04	4.28E + 06	69	VEGFSecFLS_MaxbyTGFb	1.35E + 00	7.76E + 01
5	kg_Macrophage_Baseline	4.51E + 03	7.35E + 06	70	TNFaSecFLS_MaxbyIL10	7.48E-02	5.83E + 00
6	kdiff_BCells_PlasmaCells	3.80E−06	2.63E−01	71	LeukoInflux_MaxbyCAM	1.08E−01	8.04E + 00
7	kg_Th1_Baseline	1.32E + 02	1.91E + 08	72	TCellProlif_MaxbyIL10	9.04E−02	6.37E + 00
8	kg_Th17_Baseline	3.09E−02	2.04E + 05	73	BCellApop_MaxbyBAFF	7.36E−02	4.93E + 00
9	kg_Treg_Baseline	1.38E + 03	7.24E + 08	74	TNFaSecMacro_MaxbyIL10	9.08E−02	5.78E + 00
10	F_AutoAb	8.59E−02	3.85E + 02	75	IL6SecMacro_MaxbyIL10	9.06E−02	6.07E + 00
11	F_BAFF	2.17E−02	8.79E + 01	76	MacroInflux_MaxbyTGFb	5.14E−01	2.81E + 01
12	F_CAM	1.39E−02	4.15E + 01	77	TNFaSecMacro_MaxbyAutoAb	8.32E−01	4.79E + 01
13	F_VEGF	2.19E−02	6.90E + 01	78	BCellProlif_MaxbyIL6	3.16E−01	1.82E + 01
14	F_TNFa	2.17E−02	8.38E + 01	79	BCellProlif_MaxbyIFNg	7.50E−02	5.56E + 00
15	F_TGFb	1.75E−02	5.22E + 01	80	BCellProlif_MaxbyIL10	9.89E−01	7.33E + 01
16	F_RANTES	1.39E−02	4.15E + 01	81	BCellProlif_MaxbyTGFb	1.73E−02	1.10E + 00
17	F_MIP3	3.45E−02	1.26E + 02	82	IFNgSecCTL_MaxbyIL6	5.21E−02	3.49E + 00
18	F_MCP1	1.10E−02	3.30E + 01	83	CTLProlif_MaxbyIL1b	1.68E−01	7.14E + 00
19	F_IL6	2.20E−02	6.58E + 01	84	CTLProlif_MaxbyTGFb	6.27E−02	4.20E + 00
20	F_IL23	1.73E−02	6.04E + 01	85	IFNgSecCTL_MaxbyTGFb	9.04E−02	6.37E + 00
21	F_IL1b	1.36E−02	6.41E + 01	86	VEGFSecEndo_MaxbyTGFb	3.28E−01	2.31E + 01
22	F_IL17	4.33E−02	1.75E + 02	87	MIP3SecFLS_MaxbyTNFa	1.14E + 00	7.28E + 01
23	F_IL12	1.73E−02	6.34E + 01	88	MIP3SecFLS_MaxbyIL1b	1.35E + 00	7.76E + 01
24	F_IL10	1.37E−02	5.28E + 01	89	MIP3SecFLS_MaxbyIL17	6.04E−01	3.30E + 01
25	F_IFNg	5.47E−02	2.00E + 02	90	MCP1SecFLS_MaxbyIL1b	1.19E + 00	8.00E + 01
26	F_GMCSF	1.74E−02	5.48E + 01	91	MCP1SecMacro_MaxbyIL1b	4.34E−02	2.90E + 00
27	kIn_Treg_Baseline	9.77E + 02	4.07E + 07	92	IL17SecTh17_MaxbyIL1b	1.35E + 00	7.76E + 01
28	kIn_Th1_Baseline	1.55E + 03	6.46E + 07	93	IL17SecTh17_MaxbyIL6	1.09E−01	7.29E + 00
29	kIn_Macrophage_Baseline	6.17E + 01	2.57E + 06	94	CTLApop_MaxbyTGFb	7.45E−02	3.87E + 00
30	kIn_CTL_Baseline	6.17E + 01	2.57E + 06	95	AutoAbSecBCell_MaxbyIL6	4.55E−02	2.90E + 00
31	kIn_BCells_Baseline	9.55E + 01	6.61E + 06	96	BCellDiff_MaxbyIL6	1.03E−01	5.35E + 00
32	kIn_Th17_Baseline	1.55E + 02	6.46E + 06	97	EndoProlif_MaxbyGMCSF	7.41E−02	4.27E + 00
33	kIn_Endo_Baseline	2.45E + 02	1.02E + 07	98	Endoinflux_MaxbyGMCSF	1.88E−01	1.47E + 01
34	Endoinflux_MaxbyVEGF	1.30E−01	9.66E + 00	99	TNFaSecMacro_MaxbyGMCSF	3.16E−01	1.82E + 01
35	EndoProlif_MaxbyVEGF	3.30E−01	2.10E + 01	100	CTLProlif_MaxbyIL12	6.01E−01	3.64E + 01
36	Endoinflux_MaxbyTNFa	3.15E−01	2.00E + 01	101	Th1Apop_MaxbyIL12	2.99E−02	2.11E + 00
37	EndoApop_MaxbyTNFa	1.96E−01	1.02E + 01	102	Th17Prolif_MaxbyIL23	1.08E−01	8.04E + 00
38	EndoApop_MaxbyVEGF	8.77E−02	4.34E + 00	103	IL17SecCTL_MaxbyIL23	3.18E−01	1.65E + 01
39	FLSProlif_MaxbyTNFa	1.95E−01	1.07E + 01	104	IL17SecTh17_MaxbyIL23	1.09E−01	7.66E + 00
40	Endoinflux_MaxbyIL6	1.96E−01	1.02E + 01	105	MacroProlif_MaxbyTNFa	1.09E−01	7.29E + 00
41	IL6SecFLS_MaxbyIL1b	1.43E−01	6.38E + 00	106	RANTESSecFLS_MaxbyTNFa	2.28E−01	1.45E + 01
42	MacroInflux_MaxbyIL17	1.96E−01	1.02E + 01	107	RANTESSecFLS_MaxbyIL1b	1.09E−01	7.29E + 00
43	FLSProlif_MaxbyIL1b	1.95E−01	1.07E + 01	108	RANTESSecFLS_byTNFa_MaxbyIFNg	3.30E−01	2.00E + 01
44	FLSProlif_MaxbyIL17	1.66E−01	9.55E + 00	109	RANTESSecEndo_MaxbyTNFa	1.60E + 00	7.52E + 01
45	FLSProlif_MaxbyTGFb	1.88E−01	1.40E + 01	110	RANTESSecEndo_byTNFa_MaxbyIFNg	4.58E−02	2.51E + 00
46	EndoProlif_MaxbyIL1b	2.82E−02	1.62E + 00	111	IL6SecFLS_MaxbyRANTES	8.79E−02	4.13E + 00
47	Th1Prolif_MaxbyTGFb	7.43E−02	4.06E + 00	112	TCellinflux_MaxbyRANTES	1.20E + 00	7.26E + 01
48	TCellProlif_MaxbyIL6	6.87E−01	4.60E + 01	113	RANTESSecEndo_MaxbyIL1b	4.40E−01	2.17E + 01
49	TregProlif_MaxbyTGFb	3.18E−01	1.57E + 01	114	RANTESSecFLS_byIL1b_MaxbyIFNg	2.31E−01	1.09E + 01
50	IL6SecMacro_MaxbyTNFa	2.72E−01	2.02E + 01	115	MacroInflux_MaxbyRANTES	2.40E−02	1.32E + 00
51	IL1bSecMacro_MaxbyIL17	1.08E−01	8.04E + 00	116	CTLInflux_MaxbyRANTES	1.06E−01	4.30E + 00
52	TNFaSecMacro_MaxbyIL17	1.02E−01	6.81E + 00	117	MCP1SecFLS_MaxbyTNFa	9.86E−01	7.69E + 01
53	TCellinflux_MaxbyTNFa	3.94E−01	2.78E + 01	118	FLSProlif_MaxbyIL6	2.28E−01	1.45E + 01
54	IFNgSecTh1_MaxbyIL10	7.40E−02	4.48E + 00	119	IFNgSecMacro_MaxbyIL12	1.09E−01	7.29E + 00
55	Endoinflux_MaxbyIL17	1.43E−01	6.70E + 00	120	Th1Prolif_MaxbyIL12	4.35E−01	2.77E + 01
56	Endoinflux_MaxbyTGFb	7.52E−02	5.30E + 00	121	Th17Prolif_MaxbyIL1b	8.36E−01	4.35E + 01
57	VEGFSecFLS_MaxbyIL1b	1.30E−01	9.66E + 00	122	TregProlif_MaxbyIL6	4.32E−02	3.20E + 00
58	VEGFSecFLS_MaxbyIL6	4.58E−02	2.51E + 00	123	IFNgSecCTL_MaxbyIL12	1.08E−01	8.04E + 00
59	VEGFSecFLS_MaxbyTNFa	4.55E−02	2.90E + 00	124	PlasmaProlif_MaxbyIL6	1.89E−01	1.27E + 01
60	VEGFSecFLS_MaxbyIL17	1.30E−01	1.01E + 01	125	TCellApop_MaxbyIL6	5.19E−02	3.85E + 00
61	IL6SecFLS_MaxbyIL17	7.16E−01	3.20E + 01	126	MacroApop_MaxbyTNFa	9.08E−02	5.78E + 00
62	LymphoInflux_MaxbyMIP3	7.11E−01	3.70E + 01	127	MacroApop_MaxbyIFNg	1.02E−01	6.18E + 00
63	MacroProlif_MaxbyGMCSF	2.71E−01	1.34E + 01	128	MacroApop_MaxbyGMCSF	9.08E−02	5.78E + 00
64	GMCSFSecFLS_MaxbyTNFa	6.00E−01	3.82E + 01	129	PlasmaCellApop_MaxbyIL6	1.09E−01	6.95E + 00
65	GMCSFSecMacro_MaxbyTNFa	1.08E−01	8.04E + 00				

**Fig. 13:**
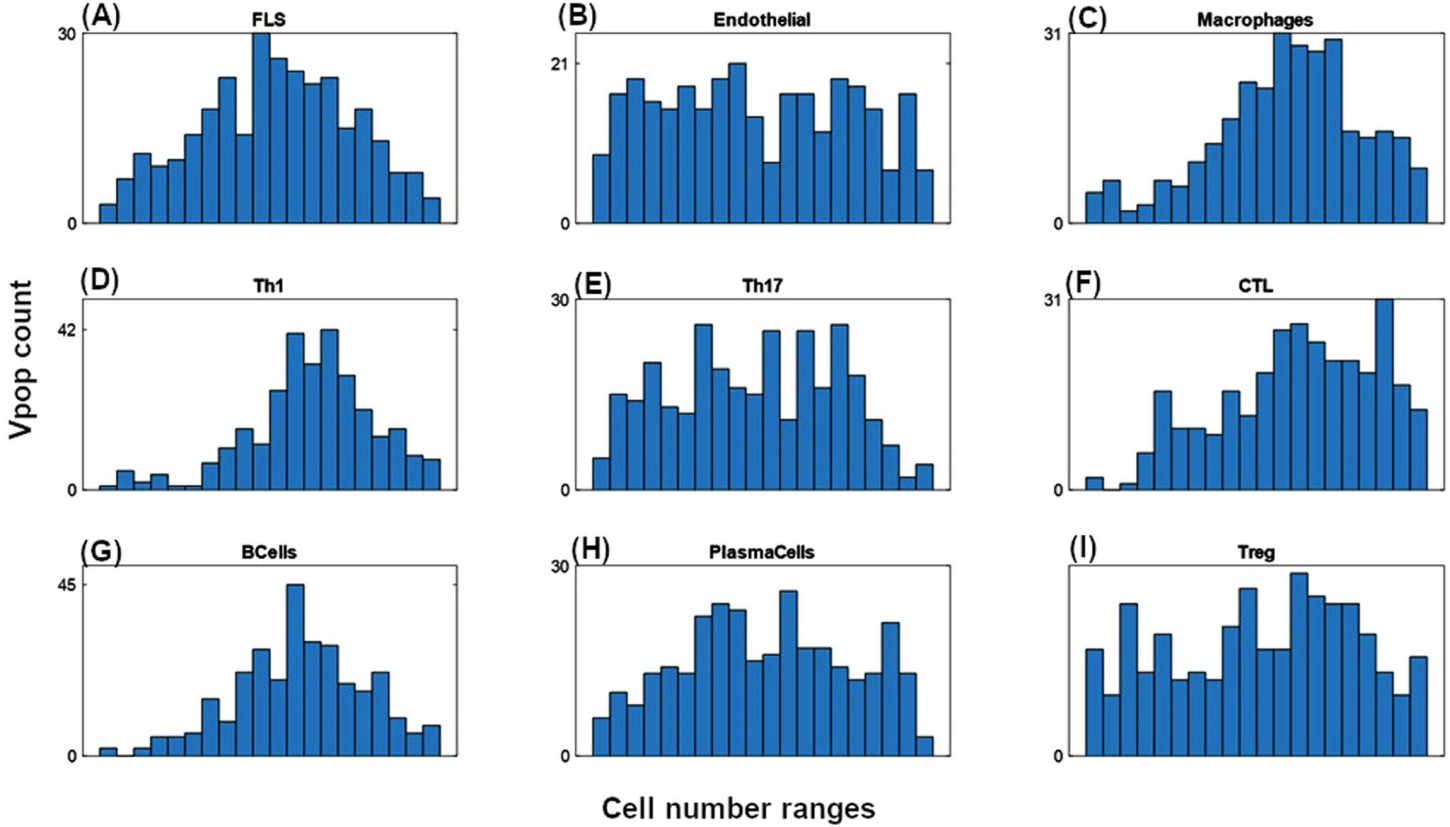
Distribution plots for the baseline cell densities of the cells in the model. Distribution plots for the Baseline Cell Densities in the Vpop for (**A**) FLS, (**B**) Endothelial cells, (**C**)Macrophages, (**D**) Th1,(**E**) Th17,(**F**) CTL, (**G**) Bcells, (**H**)Plasma cells and (**I**)Tregs respectively. The mean/ median values of these cell densities and their ranges are derived from literature (see Supplementary Data 1). The plots show that Vpop has a broad distribution of cell densities at baseline that spans the range determined from the literature. See Table 5 for the ranges for these distributions for cells.

**Fig. 14:**
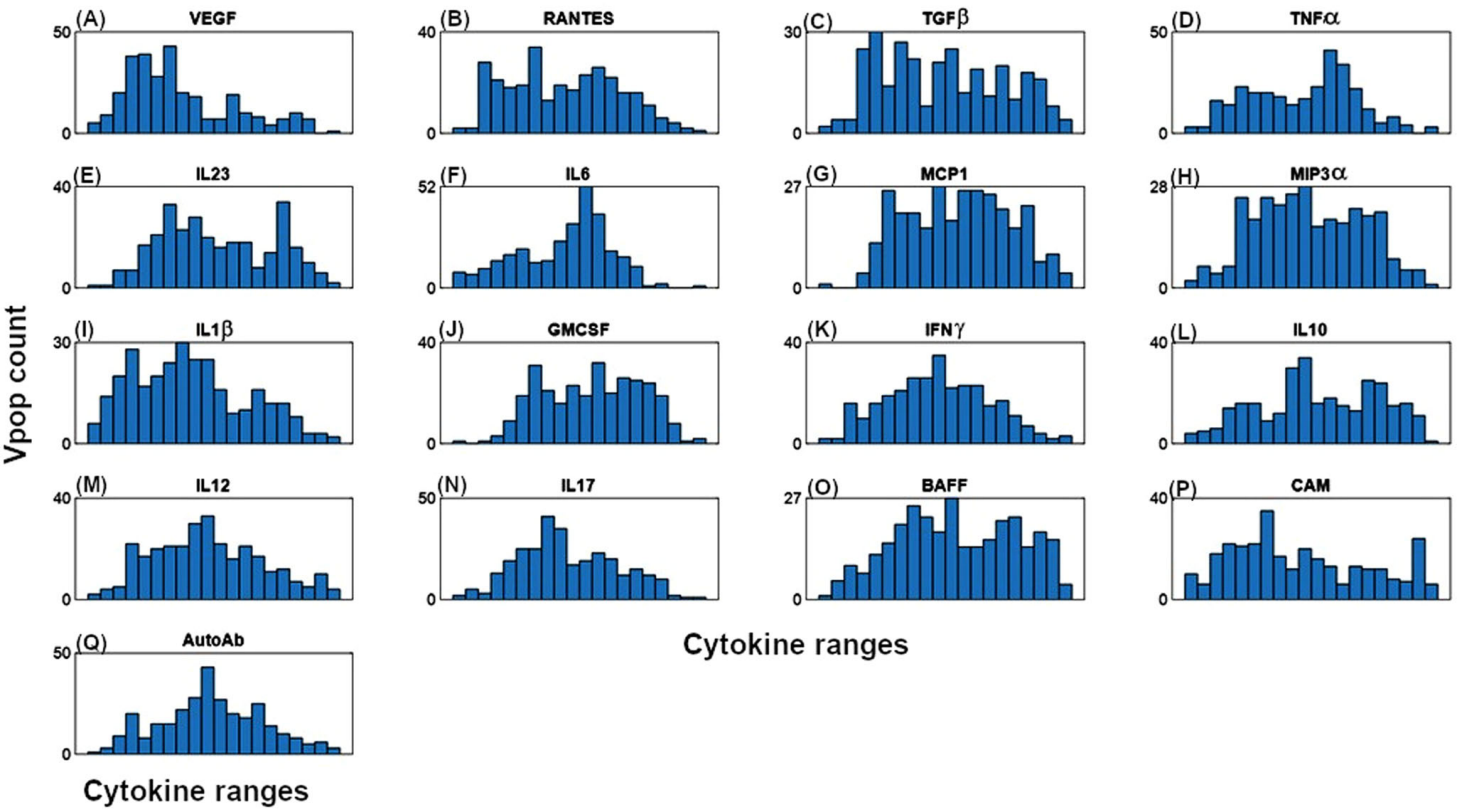
Distribution plots for baseline cytokine concentrations in the Vpop for the cytokines in the model. Distribution plots for the Baseline cytokine concentrations in the Vpop for (**A**)VEGF (**B**)RANTES (**C**) TGF-b (**D**) TNF-a (**E**) IL-23 (**F**) IL6 (**G**)MCP-1 (**H**) MIP-3a (**I**)IL-1b (**J**)GMCSF (**K**)IFN-g (**L**)IL-10 (**M**) IL-12 (**N**) IL-17 (**O**)BAFF (**P**)CAM (**Q**) AutoAb. The mean/ median values of these cytokine concentrations and their ranges are derived from literature (see Supplementary Data 1). The plots show that the Vpop has a broad distribution of concentrations at baseline that spans the range determined from the literature. See Table 6 for the ranges for these distributions for cytokines.

The table also describes the X-axis limits of the histograms.
  - The distribution of cells and cytokines in the model at steady state was also determined to show that the entire range of cell and cytokine values obtained from the literature were spanned by the Vpop, as seen in Figs. 13 and 14. Table 5 provides the log transformed ranges of the cell densities at baseline in the Vpop while Table 6 provides the log transformed ranges of cytokine concentration at baseline in the Vpop.

**Table 5:** The table describes the distribution of log transformed baseline cell density of the virtual population

ID	Cell	LB	UB	ID	Cell	LB	UB
1	FLS	1.31E + 06	1.53E + 08	6	CTL	8.38E + 02	6.56E + 06
2	Endothelial	4.13E + 07	8.31E + 07	7	Bcells	6.03E + 03	4.17E + 08
3	Macrophages	3.73E + 06	1.94E + 08	8	Plasma Cells	7.08E + 04	1.41E + 08
4	Th1	3.32E + 04	2.88E + 08	9	Tregs	3.51E + 04	9.02E + 07
5	Th17	1.64E + 03	2.02E + 07				

The table also describes the X-axis limits of the histograms.

**Table 6:** The table describes the distribution of log transformed baseline cytokine concentration of the virtual population

ID	Cytokine	LB	UB	ID	Cytokine	LB	UB
1	VEGF	1.48E-02	1.70E + 03	10	GMCSF	9.12E-05	1.74E + 01
2	RANTES	2.34E-04	2.69E + 01	11	IFNg	3.31E-06	7.59E + 02
3	TGFb	2.40E-04	1.66E + 01	12	IL10	4.90E-03	2.04E + 02
4	TNFa	5.25E-04	7.59E + 02	13	IL12	3.72E-05	4.27E + 00
5	IL23	2.40E-04	1.66E + 01	14	IL17	9.33E-07	1.70E + 01
6	IL6	9.12E-01	1.74E + 05	15	BAFF	3.80E-03	2.63E + 02
7	MCP1	2.34E-02	2.69E + 03	16	CAM	1.08E-03	5.09E + 00
8	MIP3	5.25E-05	7.59E + 01	17	AutoAb	3.16E + 01	1.26E + 08
9	IL1b	8.32E-04	1.20E + 03				

The table also describes the X-axis limits of the histograms

### Software details

The model consists of a series of ODEs representing the dynamics of the cells, cytokines and regulatory interactions represented. The general form of these ODEs is described in the section on Model reactions and in the section, “Additional information on Methods”. The model was created and simulated in MATLAB Simbiology. The virtual cohort generation and simulations were run using gQSPsim^94^. The Vpop selection was done using a genetic algorithm developed by Vantage Research. Plots were generated using MATLAB.

## Supplementary information

Supplementary Data 1

Supplementary Data 2

## Data Availability

Supplementary Data 2 contains the list of parameters obtained from the literature along with the references and calculations; other model parameters were estimated to fit the data.

## Code availability

The sbproj file along with the parameters and variants has been uploaded to git-hub. A detailed description of how to set up and run the model is also attached (User_guide.pdf).
